# Repurposed antihypertensive drugs for negative symptoms in schizophrenia: A systematic review and meta‐analysis

**DOI:** 10.1111/pcn.13892

**Published:** 2025-09-09

**Authors:** Thiago Carnaval, My Bui, Jesús Villoria, Dolores Rodríguez, José Manuel Menchón, Francisco Ciruela, Sebastián Videla

**Affiliations:** ^1^ Pharmacology Unit, Department of Pathology and Experimental Therapeutics, School of Medicine and Health Sciences, Institute of Neurosciences University of Barcelona L'Hospitalet de Llobregat Spain; ^2^ Neuropharmacology & Pain Group, Neuroscience Program Bellvitge Biomedical Research Institute L'Hospitalet de Llobregat Spain; ^3^ Department of Research, Innovation, and Learning Sant Joan de Déu Hospital Barcelona Spain; ^4^ Design and Biometrics Department Medicxact Alpedrete Madrid Spain; ^5^ Department of Pharmaceutical Sciences Utrecht University Utrecht The Netherlands; ^6^ Clinical Pharmacology Department Bellvitge University Hospital L'Hospitalet de Llobregat Spain; ^7^ Psychiatry and Mental Health Group, Neuroscience Program Bellvitge Biomedical Research Institute (IDIBELL) L'Hospitalet de Llobregat Spain; ^8^ Department of Clinical Sciences, School of Medicine University of Barcelona L'Hospitalet de Llobregat Spain; ^9^ Network Centre for Biomedical Research on Mental Health (CIBERSAM) Carlos III Health Institute (ISCIII) Madrid Spain; ^10^ Clinical Research Support Area, Clinical Pharmacology Department Germans Trias i Pujol University Hospital Barcelona Spain

**Keywords:** antihypertensive drugs, drug repurposing, psychopharmacology, psychosis

## Abstract

Schizophrenia is a complex psychiatric disorder characterized by positive, negative, and general psychopathological symptoms. While antipsychotic drugs are effective for positive symptoms, they provide limited benefit for negative symptoms, which are often persistent and strongly associated with functional disability. Additionally, up to 30% of patients exhibit resistance to current treatments, including clozapine. These challenges underscore the need for novel adjunctive strategies. This systematic review and meta‐analysis, preregistered in PROSPERO (CRD42022359199), evaluated the efficacy and safety of repurposed antihypertensive drugs (AHTs) as adjunctive treatments for schizophrenia. PubMed, Web of Science, and Scopus were searched for double‐blind, randomized controlled trials (RCTs) published since 2000. Twelve studies were included (*n* = 436; sodium nitroprusside = 6; diuretics = 4; telmisartan = 1; clonidine = 1). Meta‐analyses were conducted on Positive and Negative Syndrome Scale (PANSS) outcomes and adverse event (AE) rates. Standardized mean differences (SMDs) were calculated as Hedges' g using a restricted maximum likelihood estimator. Incidence rate ratios modeled AE rates. AHTs significantly improved negative symptoms (SMD = −0.37 [−0.59, −0.15]; *I* ^2^ = 43.8%), positive symptoms (SMD = −0.29 [−0.53, −0.06]; *I* ^2^ = 25.1%), general psychopathology (SMD = −0.28 [−0.48, −0.08]; *I* ^2^ = 0.0%), and total symptoms (SMD = −0.44 [−0.66, −0.21]; *I* ^2^ = 0.0%). No significant increase in AEs was observed. Overall, repurposed AHTs—particularly sodium nitroprusside—may offer adjunctive therapeutic benefits for schizophrenia treatments. Some diuretics also showed preliminary signals of efficacy. However, findings are preliminary and require confirmation in larger, long‐term RCTs.

Schizophrenia is a complex, severe, and disabling mental disorder characterized by positive (delusions, hallucinations, disorganized thought), negative (alogia, social withdrawal, blunted affect), and general psychopathological (guilt feelings, anxiety, mannerisms) symptoms.^1^, ^2^ The disease has been associated with up to 15 years of potential life lost, largely due to an increased suicide risk,^3^ increased prevalence of substance abuse,^4^, ^5^, ^6^, ^7^ unhealthy lifestyle,^8^, ^9^, ^10^ and pronounced rates of cardiovascular disease and diabetes.^11^, ^12^, ^13^, ^14^

Antipsychotic drugs are the cornerstone of treatment, being particularly effective in treating positive symptoms.^15^ However, their limited efficacy in addressing negative and cognitive symptoms—which are often more persistent and functionally debilitating—remain a major concern.^16^, ^17^ Negative symptoms in particular tend to be more resistant to pharmacological treatment, contribute significantly to poor social and occupational functioning, and are among the strongest predictors of long‐term disability. Additionally, treatment‐resistant schizophrenia remains a significant challenge, with a considerable subset (12–30%) failing to respond even to clozapine,^18^, ^19^ indicating a pressing need for more effective treatments.

In light of these challenges, drug repurposing seems a promising strategy, leveraging existing medications with well‐known safety and pharmacokinetic profiles for new therapeutic uses.^20^ Antihypertensive drugs (AHTs) have garnered interest for repurposing due to the bidirectional relationship between hypertension and various psychiatric disorders.^21^ For instance, angiotensin II type 1 receptor (AT_1_R) blockers (ARBs) and calcium channel blockers might be candidates for Alzheimer's disease;^22^ the loop diuretic bumetanide produced beneficial effects in cases of autism, schizophrenia, and temporal lobe epilepsy;^23^ and β blockers knowingly improve anxiety symptoms.^24^ Additionally, nitric oxide (NO) donors—particularly sodium nitroprusside (SNP)—have received growing interest following promising early clinical findings in schizophrenia patients (i.e., the rapid improvement of symptoms following a single infusion).^25^

This systematic review and meta‐analysis primarily aimed to assess the effect of repurposed AHTs on negative symptoms, given the limited efficacy of standard treatments in this domain, and to evaluate their impact on positive symptoms, general psychopathology, and total symptom severity.

## Methods

This study was conducted in accordance with the PRISMA recommendations for systematic reviews and meta‐analyses and was preregistered with PROSPERO (CRD42022359199).^26^ This meta‐analysis used publicly available data from previously published studies, therefore, ethical approval and informed consent were not required.

### Search strategy and selection criteria

PubMed®, Web of Science™, and Scopus® databases were initially searched for relevant English language randomized controlled trials (RCTs)—full search terms available in Supplementary Table 1, Supplementary Table 2, and Supplementary Table 3. Two authors (T.C. and M.B.) independently screened titles and abstracts using EndNote™ version 20.^27^ Discrepancies were settled through discussion, with a third author (S.V.) acting as referee, if necessary. In total, five records required third‐reviewer arbitration. Included studies met the following inclusion criteria: (1) double‐blind RCTs reporting the repurposing of AHTs for the treatment of schizophrenia/schizoaffective disorder as add‐on therapy to stable antipsychotic medication; (2) conducted with adult patients (≥18 years of age) of either sex; (3) placebo‐controlled; and (4) reporting on at least one component of the Positive and Negative Syndrome Scale (PANSS).

We restricted our search to studies published from 2000 onwards for different reasons. First, a 2001 Cochrane systematic review^28^ had already synthesized the earlier literature on beta‐blockers as adjunctive treatment for schizophrenia but found limited, poorly reported evidence.^28^ Second, this cut‐off aligns with the widespread adoption of modern diagnostic criteria (DSM‐IV and ICD‐10), PANSS standardization in schizophrenia trials, and the dissemination of CONSORT guidelines, which improved trial reporting quality. This time restriction aimed to ensure a focus on contemporary clinical practice and outcome assessment methods.

Studies meeting the initial eligibility criteria were read in full. The last literature search was performed on March 31, 2025.

### Outcomes, data extraction, and study quality assessment

#### Outcomes

The primary outcome measure was the standardized mean difference (SMD) in the PANSS negative symptoms subscale (N‐PANSS) score, compared to placebo.

Secondary outcome measures included:

1. Efficacy: SMDs in the total PANSS (T‐PANSS), positive symptoms (P‐PANSS), and general psychopathology (G‐PANSS) scores, all compared to placebo.
2. Safety: the incidence rate ratio (IRR) of AEs as defined and assessed in each individual study. AE definitions varied across trials, ranging from standardized tools to descriptive reporting without formal criteria (Supplementary Table 4).
3. Exploratory: SMDs in N‐PANSS, T‐PANSS, P‐PANSS, and G‐PANSS scores, compared to placebo, within each AHT class, and the SMDs in vital signs (systolic and diastolic blood pressure and heart rate) at baseline and post‐infusion for patients receiving sodium nitroprusside (SNP) or placebo.

#### Study quality assessment and data extraction

Two reviewers (T.C. and M.B.) independently assessed the risk of bias using Cochrane's RoB2 tool.^29^ Data from the included studies were extracted into separate spreadsheets and then compared, with any inconsistencies resolved through discussion. If no consensus was achieved, a third reviewer (S.V.) acted as a referee. Data extracted from each study were tabulated and included: (i) first author surname and study year; (ii) country where it was conducted; (iii) study design; (iv) diagnosis; (v) setting (in‐ or outpatients); (vi) treatment details; (vii) demographic characteristics (age and sex); (viii) illness duration; (ix) follow‐up length; and (x) primary and secondary outcomes. Two corresponding authors were contacted by email for data clarification. One responded and provided the necessary information, allowing the study to be included. The other did not respond, and due to inconsistencies between tabulated and plotted results in the original report, the study was excluded. Data from studies providing results only as plots were extracted with the WebPlotDigitizer software (version 4.6) for Windows®.^30^, ^31^

### Handling of specific trial designs

In accordance with established methodological guidelines, we applied specific strategies to ensure appropriate inclusion of studies with crossover, sequential parallel comparison design (SPCD), and multi‐arm structures. For crossover trials, only first‐period data were included to minimize the risk of carryover effects.^32^ In studies using SPCD, we restricted our analyses to first phase data to avoid potential bias from the enriched design of the second phase and maintain consistency with standard parallel‐group RCTs. For trials with multiple treatment arms sharing a common placebo group, the placebo sample size was evenly split between comparisons to prevent unit‐of‐analysis errors in pairwise meta‐analyses.^32^ These methodological choices were aimed at preserving the integrity and comparability of effect estimates across included studies.

### Statistical analyses

Random effects meta‐analyses were conducted for the T‐PANSS and its subscales (P‐PANSS, N‐PANSS, and G‐PANSS) using SMDs calculated as Hedges' g, along with 95% confidence intervals (CIs) were calculated. A restricted maximum likelihood (REML) estimator was applied to account for between‐study heterogeneity. Wald‐type CIs were used for inference, and prediction intervals were calculated to assess the dispersion of true effects. Subgroup analyses were performed by drug class using the same approach.

When means were not reported, they were estimated *via* a weighted average of the median, minimum, and maximum values, while standard deviations (SD) were calculated as one‐quarter of the range.^33^ For studies reporting standard errors (SE) or CIs, SDs were back‐calculated using the delta method or CI width divided by the appropriate t‐value.^32^, ^33^ Mean changes from baseline and their associated SDs were calculated (when not directly provided) for each group and PANSS subscale. Where individual‐level correlations were unavailable, sensitivity analyses were performed assuming correlation coefficients (*r*) of 0.3, 0.5, and 0.8 to model change‐score variances (Supplementary Table 1), although we selected *r* = 0.5 as a moderate and widely accepted default estimate in the absence of study‐specific data.^32^

AE rates were modeled using IRRs based on the number of events and sample size per study arm, and assuming equal exposure times. Log‐transformed IRRs were calculated (to stabilize the variance) with a continuity correction of 0.5 for studies reporting zero events. These were pooled using a REML estimator and back‐transformed to obtain summary IRRs with 95% CIs and prediction intervals.

Heterogeneity was quantified using the *I* ^2^ statistic and the between‐study variance τ^2^. Leave‐one‐out sensitivity analyses were conducted for all PANSS outcomes to evaluate the influence of each study on the overall effect. In addition, a separate sensitivity analysis was performed by restricting the meta‐analysis to studies rated as having a low risk of bias. Publication bias was evaluated using Egger's test and contour‐enhanced funnel plots.

To address multiple testing across PANSS subscales, adjusted *P*‐values were computed using Holm's method, a stepwise procedure that controls the family‐wise error rate while maintaining greater statistical power than the more conservative Bonferroni correction.

All analyses were performed with the *meta* and *metafor* packages in R, version 4.2.3 (R Foundation for Statistical Computing, Vienna, Austria).^34^, ^35^, ^36^

## Results

### Description of included studies

**Fig. 1:**
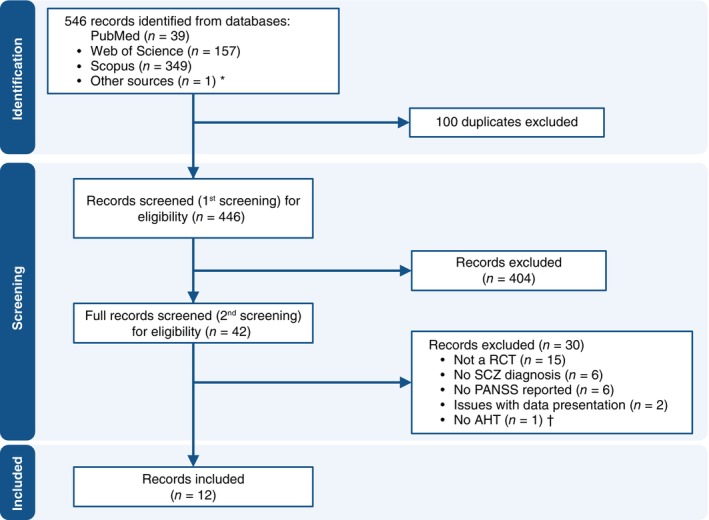
Study selection process. RCT, randomized controlled trial; SCZ, schizophrenia; PANSS, Positive and Negative Syndrome Scale; AHT, antihypertensive drugs. * EU Clinical Trials Register. † This study used a T‐type calcium channel antagonist that has been specifically developed as a central nervous system agent, which is not classified as an antihypertensive drug.

The literature search identified 546 records. After removing duplicates and screening titles and abstracts, 43 full‐text articles were assessed for eligibility. Of these, 30 were excluded (Supplementary Table 5), and 12 unique studies were ultimately included in the meta‐analysis. One study with a three‐arm design was considered twice in the analyses (with the placebo group split), resulting in 13 study comparisons overall (Fig. 1).

Details of the included studies are presented in Table 1, while the eligibility criteria specific to each study are summarized in Supplementary Table 6. Notably, two studies were excluded due to issues with data presentation. One study showed discrepancies between the data reported in tables and the values extracted from figures,^37^ while the other failed to specify whether error bars represented SDs, SEs, or 95% CIs.^38^

**Table 1:** Characteristics of the included studies

Study (Year)	Country	Design	Diagnosis/Setting	DM/HT Exclusion Criteria	Groups	*n*	Sex (M/F)	Mean (SD) Age, years	Mean (SD) Illness Duration, years	Follow‐up, weeks
Hallak *et al*. (2013)^25^	BR	DB RCT, Parallel	SCZ/Inpatients	NR	SNP 0.5 μg/kg/min × 4 h; single IV infusion	10	7/3 7/3	25.5 (6.7) 25.6 (3.9)	2.9 (2.3)	4
					Pb	10			3.2 (2.7)	
Stone *et al*. (2016)^40^	UK	DB RCT, Parallel	SCZ and SCZAf/Outpatients	NR	SNP 0.5 μg/kg/min × 4 h; single IV infusion	10	7/3	34.0 (9.0)	12.0 (7.0)	4
					Pb	10	8/2	40.0 (10.0)	17.0 (8.0)	
Wang *et al*. (2018)^41^	CN	DB RCT, Parallel	SCZ/Inpatients	NR	SNP 0.5 μg/kg/min × 4 h; 2 infusions, 1 wk. apart	21	11/10	30.5 (7.3)	7.5 (5.0)	4
					Pb	21	12/9	29.4 (7.5)	8.9 (6.6)	
Brown *et al*. (2019)^16^	US	DB RCT, SPCD	SCZ/Outpatients	NR	SNP/SNP †	18	14/4	47.1 (10.5)	NR	4
					Pb/SNP †	16	12/4	45.9 (12.3)	NR	
					Pb/Pb †	18	14/4	40.4 (11.0)	NR	
Weiser *et al*. (2020)^42^	MD/RO	DB RCT, Parallel	SCZ/Either	NR	SNP 0.5 μg/kg/min × 4 h; single IV infusion	10	7/3	36.1 (5.3)	NR	4
					Pb	10	3/7	34.5 (3.6)	NR	
Nani *et al*. (2024)^39^	BR	DB RCT, Crossover	SCZ/Outpatients	NR	SNP 0.5 μg/kg/min × 4 h; once/mo. × 4 mo.	8	7/1	30.0 (5.0)	NR	32
					Pb	7	6/1	33.0 (5.0)	NR	
Akhondzadeh *et al*. (2002)^43^	IR	DB RCT, Parallel	SCZ/Inpatients	Both *†	DZX 200 mg/day PO	21	27/15	NR (18.0–42.0)*	NR	8
					Pb	21			NR	
Rahmanzadeh *et al*. (2016)^45^	IR	DB RCT, Parallel	SCZ/NR	NR	BUME 1 mg/12h PO	14	NR	NR	NR	12
					Pb	12	NR	NR	NR	
Zandifar *et al*. (2021)^46^	IR	DB RCT, Parallel	SCZ/NR	DM *†	SPIRO 100 mg/day PO × 8 wks.	20	12/8	38.0 (9.3)	9.3 (4.1)	8
					Pb	20	11/9	40.0 (8.5)	9.6 (3.7)	
Hasan *et al*. (2023)^44^	DE	DB RCT, Parallel	SCZ/Either	NR	SPIRO 100 mg/day PO × 3 wks.	30	9/21	36.5 (20.0–62.0)*	6.0 (0.6–36.0)*	9
					SPIRO 200 mg/day PO × 3 wks.	28	9/19	35.5 (19.0–57.0)*	9.5 (0.7–27.0)*	
					Pb	26	5/21	35.5 (18.0–58.0)*	9.5 (0.9–38.0)*	
Fan *et al*. (2017)^47^	US	DB RCT, Parallel	SCZ and SCZAf/Outpatients	DM *†	TEL 40 mg/day × 2 wks., then 80 mg/day × 10 wks.	22	18/4	41.5 (12.3)	NR	12
					Pb	21	16/5	44.4 (11.5)	NR	
Kruiper *et al*. (2023)^48^	NL	DB RCT, Parallel	SCZ/Either	NR	CLON 50 μg once daily.	16	14/2	36.3 (9.9)	11.5 (67.4)†	6
					Pb	16	14/2	37.6 (6.9)	15.6 (6.7)	

DM, diabetes mellitus; HT, hypertension; M, male; F, female; SD, standard deviation; NR, not reported; IR, Iran; BR, Brazil; UK, United Kingdom; US, United States of America; CN, China; MD, Moldova; RO, Romania; DE, Germany; NL, The Netherlands; DB, double‐blind; RCT, randomized controlled trial; SPCD, sequential parallel comparison design; SCZ, schizophrenia; SCZAf, schizoaffective disorder; Pb, placebo; SNP, sodium nitroprusside; DZX, diazoxide; BUME, bumetanide; SPIRO, spironolactone; TEL, telmisartan; CLON; clonidine; mo., month; wk., week.

* Studies providing median (range) values instead of mean (SD). Of note, *Akhondzadeh et al*. provided neither mean nor median age values, but did provide the range.

^†^ The SNP/SNP group received SNP in phase 1 and SNP in phase 2 for the purpose of blinding, but the data from phase 2 were not included in the results. The Pb/SNP group received placebo in phase 1 and SNP in phase 2. The Pb/Pb group received placebo in both phases. SNP was infused at a rate of 0.5 μg/kg/min for 4 h.

^*†^ Zandifar *et al*. (2021) excluded patients with drug‐ or insulin‐dependent diabetes were excluded; Fan *et al*. (2017) excluded patients with diabetes receiving insulin and those undergoing treatments that could result in potential drug–drug interactions with telmisartan.

Ten (83.3%) studies were parallel‐group double‐blind RCTs, including one with a three‐arm design. One (8.3%) study employed a sequential comparison parallel design (SPCD), and another (8.3%) was a crossover trial. All studies investigated antihypertensive drugs administered as adjunctive treatments to stable antipsychotic regimens. Six (50.0%) studies used SNP, a nitric oxide (NO) donor,^16^, ^25^, ^39^, ^40^, ^41^, ^42^ four (33.3%) used a diuretic (either diazoxide, bumetanide, or spironolactone),^43^, ^44^, ^45^, ^46^ one (8.3%) used an ARB (telmisartan),^47^ and one (8.3%) used clonidine, an α_2_‐agonist.^48^

Sample sizes ranged from 15 to 84 participants, totaling 436 patients diagnosed with either schizophrenia or schizoaffective disorder. The mean (SD) age was 36.6 (10.4) years. Among the 410 patients with reported sex data, 171 (39.8%) were female and 259 (60.2%) were male; one study (*n* = 26) did not report sex distribution.^45^ The weighted mean (SD) follow‐up period was 8.1 (5.3) weeks, and the mean (SD) duration of illness was 10.8 (18.4) years.

### Risk of bias assessment

**Fig. 2:**
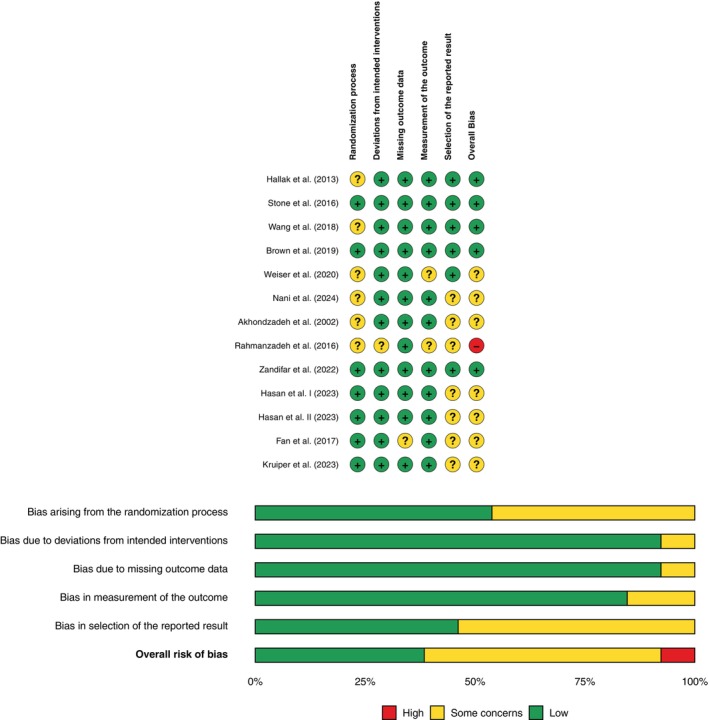
Risk of bias assessment results. Green: low risk of bias, yellow: some concerns, red: high risk of bias.

According to the RoB2 assessment, five RCTs were rated as having a low overall risk of bias.^16^, ^25^, ^40^, ^41^, ^46^ Six RCTs raised some concerns,^39^, ^42^, ^43^, ^44^, ^47^, ^48^ while one had a high overall risk (Fig. 2).^45^ Notably, although none of the individual domains in this study were rated as “high risk”, the accumulation of concerns across critical domains, particularly due to poor reporting, substantially reduces confidence in the estimated effect.

### Results of individual studies

PANSS‐related outcomes reported across the included studies are summarized in Supplementary Table 7. Six studies reported complete PANSS data, including total and all three subscale scores, at both baseline and end‐of‐trial (EoT). These trials evaluated a range of antihypertensive drugs, namely diazoxide,^43^ bumetanide,^45^ telmisartan,^47^ spironolactone,^44^, ^46^ and clonidine.^48^ Notably, although Fan *et al*. did not report the EoT scores directly, they did provide the mean (SD) changes from baseline.^47^

The remaining six studies assessed the effects of SNP infusion on schizophrenia symptoms. Of these, two reported only the subscale scores at baseline and EoT; one reported the T‐PANSS, P‐PANSS, and N‐PANSS scores; another informed only T‐PANSS and N‐PANSS scores; one provided data on P‐PANSS and N‐PANSS only; and one reported the N‐PANSS score alone. Importantly, *Weiser et al*. reported neither the EoT score nor the change from baseline for the P‐PANSS and G‐PANSS.^42^

Only three studies provided the mean (SD) change from baseline.^16^, ^46^, ^47^ This outcome measure was calculated for those that did not.

### Meta‐analysis

As described in the *Methods* section, the crossover trial was analyzed using only first‐period data,^39^ and the SPCD trial was limited to first‐phase data.^16^ In the multi‐arm study,^44^ the placebo group was appropriately split between comparisons to prevent unit‐of‐analysis errors, and the study was included twice accordingly. The GRADE summary of findings is provided in Table 2.

**Table 2:** GRADE summary of findings

Outcome	Number of Studies	*n*	Certainty of the Evidence	Effect*	95% CI	Comments
N‐PANSS	12	436	●●●○ *Moderate*	−0.37	−0.59 to −0.15	Downgraded once for risk of bias (six studies with some concerns, one with high risk).
P‐PANSS	11	416	●●●○ *Moderate*	−0.29	−0.53 to −0.06	Downgraded once for risk of bias (six studies with some concerns and one with high risk).
G‐PANSS	10	401	●●●○ *Moderate*	−0.28	−0.48 to −0.08	Downgraded once for risk of bias (five studies with some concerns and one with high risk).
T‐PANSS	8	339	●●●○ *Moderate*	−0.44	−0.66 to −0.21	Downgraded once for risk of bias (five studies with some concerns; one with high risk). Although Egger's test could not be performed due to the limited number of studies, no other signs of publication bias were detected.
Adverse Events	10	368	●●○○ *Low*	0.82	0.56 to 1.20	Downgraded once for risk of bias (5/10 showed some concerns) and once for imprecision (CI includes null; wide PI).

*Note*: N‐PANSS, positive and negative syndrome scale negative symptoms subscale score; P‐PANSS, positive and negative syndrome scale positive symptoms subscale Score; G‐PANSS, positive and negative syndrome scale general psychopathology subscale score; T‐PANSS, positive and negative syndrome scale total score.

* For PANSS Domains: Hedges g (SMD); for adverse events: incidence rate ratio (IRR).

The certainty of evidence for all PANSS outcomes was rated as moderate according to the GRADE framework. This rating reflects a single level of downgrading due to concerns about the overall risk of bias (Fig. 2). No additional downgrades were applied to the PANSS outcomes. Imprecision was not considered serious, as CIs excluded the null and sample sizes exceeded 300 participants. Inconsistency was low to moderate (*I* ^2^ ranging from 0% to 43.8%), and all outcomes showed directionally consistent effects. Publication bias was not suspected based on funnel plots (Supplementary Fig. 1) and Egger's tests (Supplementary Table 8). Although Egger's test could not be performed for T‐PANSS due to the limited number of studies, the consistency and magnitude of effects did not raise concerns. Indirectness was not considered serious, as the PANSS and adverse event data directly addressed the research question using validated and clinically relevant measures.

For AEs, the certainty of evidence was rated as low, reflecting two levels of downgrading: one for risk of bias (as half of the included studies were rated as presenting some concerns) and one for imprecision. The pooled IRR showed a wide 95% CI that included the null, and the prediction interval indicated substantial uncertainty around the estimate.

#### Negative symptoms

**Fig. 3:**
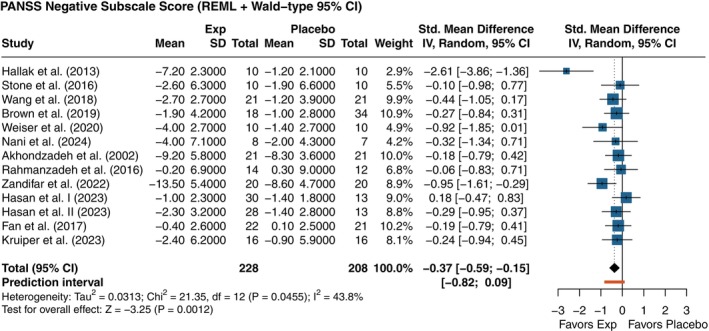
Forest plot of the effects of antihypertensive drugs on negative symptoms (N‐PANSS). Negative values favor the experimental (Exp) group. *P*‐values displayed in the forest plot are unadjusted. Adjusted *P*‐values for multiple testing using Holm's correction are presented in Supplementary Table 9.

Twelve studies (*n* = 436; 228 experimental, 208 placebo) reported changes in negative symptom severity using the N‐PANSS subscale, yielding a total of 13 comparisons. Pooled analysis showed a small‐to‐moderate, statistically significant (*P =* 0.001) effect favoring intervention over placebo (SMD [95% CI] = −0.37 [−0.59; −0.15]) (Fig. 3). Moderate heterogeneity was observed (*I* ^2^ = 43.8%), suggesting some variation in effect size across studies.

#### Positive symptoms

**Fig. 4:**
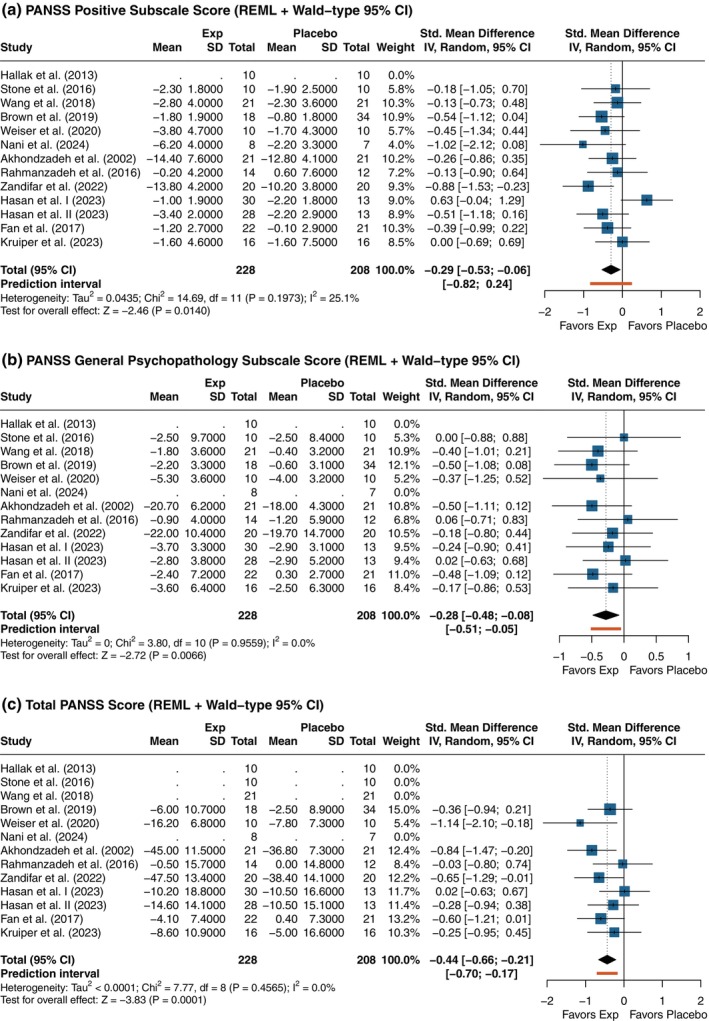
Forest plots of the effects of antihypertensive drugs on positive (P‐PANSS) and general psychopathology (G‐PANSS) symptoms, and overall disease severity (T‐PANSS). (a) Positive symptoms subscale (P‐PANSS); (b) General psychopathology subscale (G‐PANSS); (c) Total PANSS score (T‐PANSS). Negative values favor the experimental (Exp) group. *P*‐values displayed in the forest plots are unadjusted. Adjusted *P*‐values for multiple testing using Holm's correction are presented in Supplementary Table 9.

Eleven studies reported on P‐PANSS scores, yielding a total of 12 comparisons. The meta‐analysis showed a marginal, statistically detectable (*P =* 0.014) reduction in positive symptoms favoring intervention (SMD [95% CI] = −0.29 [−0.53; −0.06]) (Fig. 4a). Heterogeneity was low (*I* ^2^ = 25.1%).

#### General psychopathology symptoms

Ten studies were included in the analysis of G‐PANSS scores (11 comparisons). The pooled estimate indicated a small, statistically significant (*P =* 0.007) benefit in favor of the experimental group (SMD [95% CI] = −0.28 [−0.48; −0.08]) (Fig. 4b). Heterogeneity was negligible (*I* ^2^ = 0.0%), suggesting consistent effects across studies.

#### Global symptom severity

Eight studies contributed to the analysis of global symptom severity (nine comparisons). A moderate and statistically significant (*P =* 0.001) reduction in T‐PANSS scores was observed in the experimental group compared to placebo (SMD [95% CI] = −0.44 [−0.66; −0.21]) (Fig. 4c). No heterogeneity was detected (*I* ^2^ = 0.0%), supporting the consistency of treatment effects.

After applying Holm's correction for multiple comparisons (Supplementary Table 9), the effects of AHTs on all PANSS domains remained significant. The adjusted *p*‐values for N‐PANSS, P‐PANSS, G‐PANSS, and T‐PANSS were 0.003, 0.014, 0.013, and 0.001, respectively, supporting a consistent treatment effect across negative, positive, general psychopathology, and total symptoms.

### Safety

#### Incidence rate ratios of adverse events

**Fig. 5:**
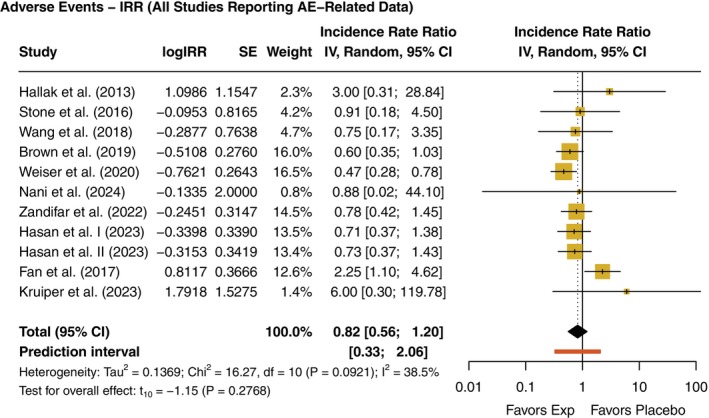
Forest plots of pooled incidence rate ratios (IRRs) of adverse events. IRR, incidence rate ratio; Exp, experimental; Pb; placebo. IRR Values <1 favor the experimental (Exp) group. *P*‐values displayed in the forest plot are unadjusted. Adjusted *P*‐values for multiple testing using Holm's correction are presented in Supplementary Table 10.

Regarding safety, the IRR of AEs was assessed across 10 studies,^16^, ^25^, ^39^, ^40^, ^41^, ^42^, ^44^, ^46^, ^47^, ^48^ yielding a total of 11 comparisons. The pooled IRR (95% CI) was 0.82 (0.57; 1.18), indicating no clear difference in the rate of AEs between patients receiving AHTs and those on placebo. While studies such as *Weiser et al*.^42^ and *Brown et al*.^16^ showed a trend toward fewer AEs in the experimental group, only the former featured an IRR with a CI excluding the null. Conversely, *Fan et al*.^47^ showed a higher incidence of AEs in the experimental arm (IRR [95% CI] = 2.25 [1.10; 4.62]). The remaining studies yielded IRRs with wide CIs crossing the null, reflecting limited power and between‐study variability (Fig. 5). Overall, the evidence does not suggest a consistent safety signal associated with the use of antihypertensives as adjunctive treatments in schizophrenia.

#### Vital signs

**Fig. 6:**
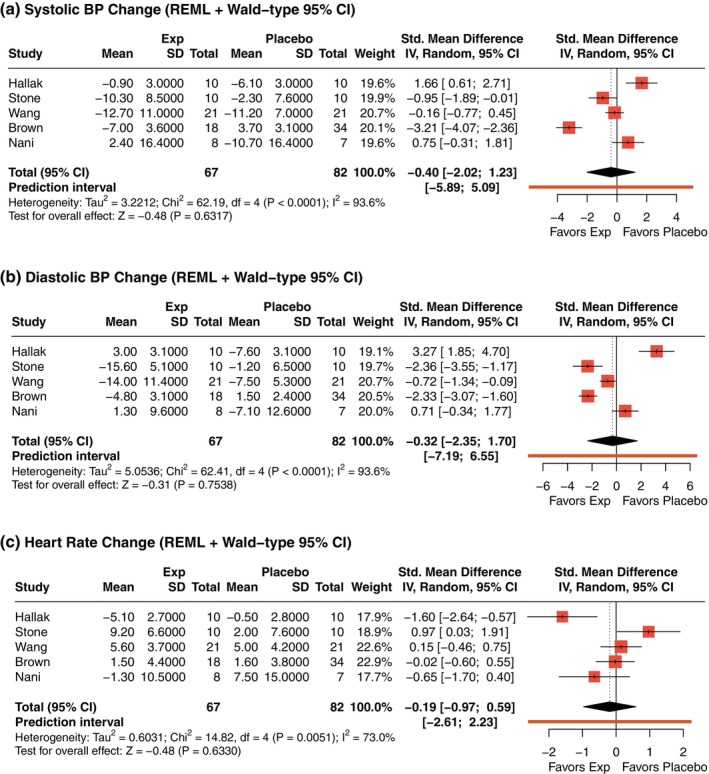
Effects of sodium nitroprusside (SNP) on vital signs during infusion. (a) Systolic blood pressure (BP); (b) Diastolic BP; (c) Heart rate. Negative SMDs reflect greater reductions in BP in the SNP group, favoring placebo in terms of hemodynamic stability. Heart rate changes may reflect compensatory responses; thus, positive SMDs favor placebo. *P*‐values displayed in the forest plots are unadjusted. Adjusted *P*‐values for multiple testing using Holm's correction are presented in Supplementary Table 10.

Regarding cardiovascular safety during SNP infusion, pooled analyses of vital sign changes showed no reliable differences between groups. The SMD in systolic blood pressure change was SMD (95% CI): −0.40 (−2.02; 1.23) (Fig. 6a), with substantial heterogeneity (*I* ^2^ = 93.6%). Diastolic pressure also showed no consistent effect (SMD [95% CI] = −0.32 [−2.35; 1.70]) (Fig. 6b), and heterogeneity remained high (*I* ^2^ = 93.6%). Similarly, no reliable effect was evidenced for the heart rate (SMD [95% CI] = −0.19 [−0.97; 0.59]) (Fig. 6c), with considerable heterogeneity (*I* ^2^ = 73.0%). Taken together, these findings suggest no consistent impact of treatment on short‐term cardiovascular parameters, though variability across studies was high.

Holm's correction confirmed that none of the observed differences in vital signs during SNP infusion were significant (all adjusted *P*‐values = 1.000; Supplementary Table 10).

### Subgroup analysis

To explore potential differences in treatment effects across pharmacological classes, subgroup analyses were conducted based on the type of AHT evaluated. *Fan et al*.^47^ and *Kruiper et al*.^48^ were excluded from these analyses, as each was the sole representative of their respective drug classes.

#### SNP only

**Fig. 7:**
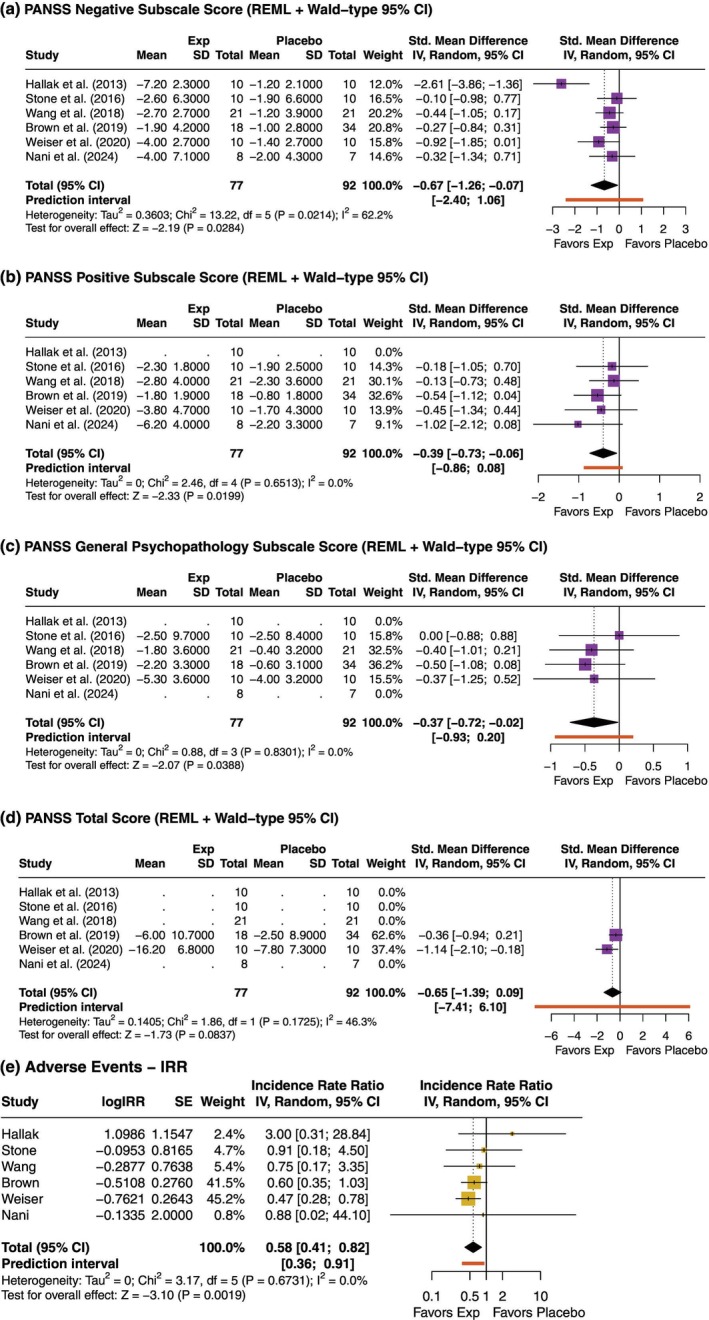
Subgroup analysis for sodium nitroprusside (SNP) trials. (a) PANSS Negative Subscale (N‐PANSS); (b) Positive Subscale (P‐PANSS); (c) General Psychopathology Subscale (G‐PANSS); (d) Total PANSS Score (T‐PANSS); (e) Incidence Rate Ratio (IRR) of adverse events. Negative SMDs favor the SNP group. For IRR, values <1 indicate fewer adverse events with SNP. P‐values displayed in the forest plots are unadjusted. Adjusted *P*‐values for multiple testing using Holm's correction are presented in Supplementary Table 9 and Supplementary Table 10.

In the SNP subgroup, an improvement in negative symptoms was observed (SMD [95% CI] = −0.67 [−1.26; −0.07]), with moderate heterogeneity (*I* ^2^ = 62.2%) (Fig. 7a). Positive symptoms also showed a reduction favoring the intervention (SMD [95% CI] = −0.39 [−0.73; −0.06]), with negligible heterogeneity (*I* ^2^ = 0%) (Fig. 7b). A similar pattern was observed for general psychopathology (SMD [95% CI] = −0.37 [−0.72; −0.02]), with *I* ^2^ = 0% (Fig. 7c). A trend toward improvement in total symptoms was noted (SMD [95% CI] = −0.65 [−1.39; 0.09]), although the possibility of no effect could not be ruled out (Fig. 7d). In terms of safety, the pooled IRR for AEs favored the SNP group (IRR [95% CI]: 0.58 [0.41; 0.82]) (Fig. 7e).

Holm's correction attenuated the strength of most PANSS‐related findings in the SNP subgroup (Supplementary Table 9). None of the associations remained below the conventional threshold for statistical significance after adjustment; for example, the effect on P‐PANSS yielded an adjusted *P*‐value of 0.080. These results should therefore be interpreted cautiously in the context of multiple testing.

In contrast, the observed reduction in AEs associated with SNP infusion remained robust following Holm's correction (adjusted *P =* 0.006; Supplementary Table 10), lending further support to a potential safety advantage in this subgroup.

#### Diuretics only

**Fig. 8:**
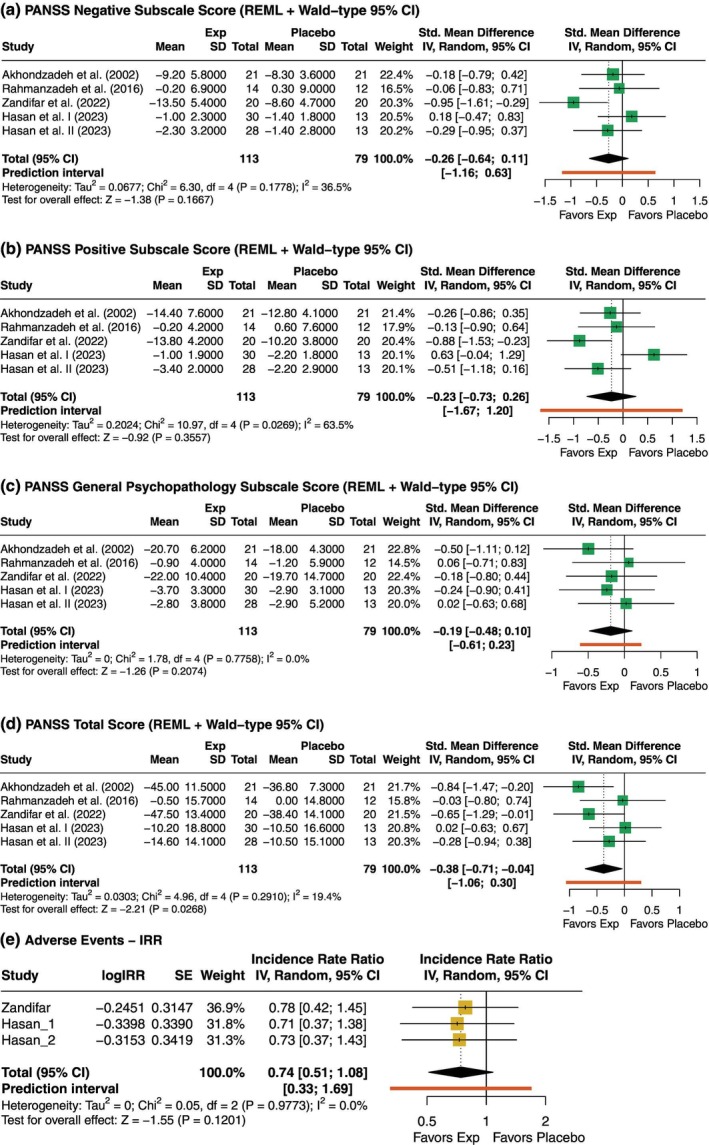
Subgroup analyses for diuretic trials. (a) PANSS Negative Subscale (N‐PANSS); (b) Positive Subscale (P‐PANSS); (c) General Psychopathology Subscale (G‐PANSS); (d) Total PANSS Score (T‐PANSS); (e) Incidence Rate Ratio (IRR) of adverse events. Negative SMDs favor the diuretic group. For IRRs, values <1 indicate fewer adverse events with diuretics. *P*‐values displayed in the forest plots are unadjusted. Adjusted *p*‐values for multiple testing using Holm's correction are presented in Supplementary Tables 9 and 10.

In the diuretic subgroup, the pooled effect for negative symptoms no clear difference between groups (SMD [95% CI] = −0.26 [−0.64; 0.11]), with moderate heterogeneity (*I* ^2^ = 36.5%) (Fig. 8a). Similarly, no clear difference was observed for positive symptoms (SMD [95% CI] = −0.23 [−0.73; 0.26]), with substantial heterogeneity (*I* ^2^ = 63.5%) (Fig. 8b). The general psychopathology subscale also showed no meaningful difference between groups (SMD [95% CI] = −0.19 [−0.48; 0.10]), with no heterogeneity (*I* ^2^ = 0%) (Fig. 8c). However, a small‐to‐moderate reduction in total symptoms was observed (SMD [95% CI] = −0.38 [−0.71; −0.04]), with low heterogeneity (*I* ^2^ = 19.4%) (Fig. 8d). As for safety, the pooled IRR suggested a potential reduction in AEs with diuretics (IRR [95% CI]: 0.74 [0.51; 1.08]) (Fig. 8e); however, the possibility of no effect cannot be ruled out.

Although the T‐PANSS initially showed a nominal association (unadjusted *P =* 0.027), the adjusted *P*‐value following Holm's correction (*P =* 0.107) exceeded the conventional threshold for significance (Supplementary Table 9. The remaining adjusted *P*‐values also indicated no detectable effects across PANSS domains, suggesting that findings in the diuretic subgroup should be interpreted with caution when accounting for multiple comparisons.

### Sensitivity analyses

**Fig. 9:**
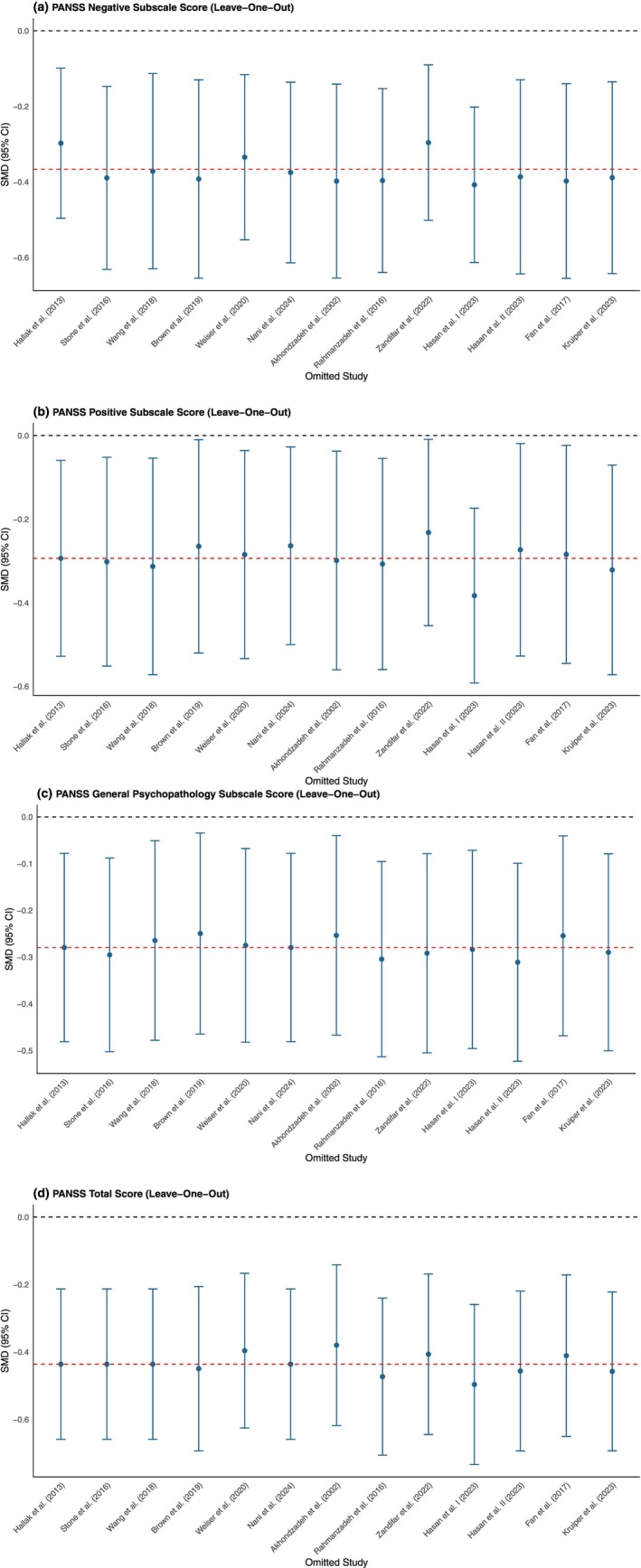
Leave‐one‐out sensitivity analyses for PANSS outcomes. (a) Negative symptoms (N‐PANSS); (b) Positive symptoms (P‐PANSS); (c) General psychopathology (G‐PANSS); (d) Total PANSS score (T‐PANSS). Each point represents the pooled standardized mean difference (SMD) and 95% CI after excluding one study at a time. The red dashed line indicates the overall pooled SMD.

Leave‐one‐out sensitivity analyses were performed to assess the robustness of the pooled effects for each PANSS outcome. For N‐PANSS, the pooled effect remained consistent across all iterations, with SMDs ranging from −0.30 to −0.41. The exclusion of individual studies did not substantially alter the overall estimate, indicating high consistency of the pooled effect (Fig. 9a).

For P‐PANSS, the results also proved stable, with SMDs ranging from −0.23 to −0.38. While omitting *Brown et al*.,^16^ *Zandifar et al*.,^46^ or *Nani et al*.^39^ brought the lower bound of the CI closer to zero, the overall pattern of effect remained consistent with the reference estimate (Fig. 9b).

In the case of G‐PANSS, all leave‐one‐out SMDs remained close to the reference estimate, ranging from −0.25 to −0.31. The exclusion of any single study did not meaningfully affect the precision or direction of the effect, further supporting the robustness of the findings (Fig. 9c).

For T‐PANSS, point estimates ranged from −0.38 to −0.50, and all corresponding CIs excluded the null value. Despite some variability across models, all iterations were consistent with the reference estimate, indicating strong overall consistency in the direction and magnitude of the T‐PANSS effect (Fig. 9d).

When restricting the analysis to studies at low risk of bias, AHTs remained superior to placebo across all PANSS domains (Supplementary Fig. 1). The SMD (95% CI) for the N‐PANSS was −0.77 (−1.49; −0.04), *P =* 0.039, *I* ^2^ = 70.9%; for the P‐PANSS: −0.45 (−0.80; −0.11), *P =* 0.010, *I* ^2^ = 6.7%; for the G‐PANSS: −0.32 (−0.64; 0.01), *P =* 0.056, *I* ^2^ = 0.0%; and for the T‐PANSS: −0.49 (−0.92; −0.06), *P =* 0.025, *I* ^2^ = 0.0%.

### Publication bias

The risk of publication bias was assessed using contour‐enhanced funnel plots (Supplementary Fig. 2) and Egger's test (Supplementary Table 7). No evidence of publication bias was detected based on these analyses. Although mild asymmetries were visually noted in some funnel plots (e.g., N‐PANSS and G‐PANSS), the corresponding Egger's test results (*P* > 0.05) suggest they are more likely attributable to sampling variability or between‐study heterogeneity rather than to systematic bias. Egger's test could not be performed for T‐PANSS due to the small number of studies reporting this outcome.

## Discussion

This systematic review and meta‐analysis combined data from 12 RCTs involving 436 participants to examine the potential of repurposing AHTs as adjunctive treatments in schizophrenia and schizoaffective disorder. Overall, the results suggest that AHTs may provide modest but consistent symptomatic benefits when added to antipsychotic therapy. However, the results should be interpreted with caution given the exploratory nature of the evidence available and the limited sample sizes.

The most consistent and clinically relevant finding was the significant improvement in negative symptoms, as captured by the N‐PANSS (*P =* 0.001), the primary outcome of this analysis. Despite their modest magnitude, these improvements are important in the context of a condition where negative symptoms remain largely refractory to current treatments.^16^, ^17^ Given their association with functional impairment and long‐term disability, even small therapeutic gains in this domain could translate into meaningful benefits for some patients.

Pooled estimates for G‐PANSS (*P =* 0.007) and T‐PANSS (*P =* 0.001) also showed significant improvements favoring AHTs over placebo, whereas the estimate for P‐PANSS (*P =* 0.014) indicated only a marginal association. The consistency of these effects across different PANSS subscales and total scores—reinforced by low heterogeneity and robust sensitivity analyses—supports the overall findings. However, the modest effect sizes and short follow‐up periods across all symptom domains underscore the preliminary nature of these findings, and further research is warranted to validate these preliminary results and determine their clinical relevance.

Subgroup analyses provided further insights into the potential heterogeneity of treatment effects by drug class. Trials conducted with SNP showed more pronounced effects across symptom domains, including reductions in both positive and negative symptoms. One plausible mechanism involves modulation of N‐methyl‐D‐aspartate receptor (NMDAR) function through NO signaling, which may partially restore glutamatergic transmission in brain regions implicated in schizophrenia (prefrontal cortex and hippocampus).^17^, ^49^, ^50^ Improvements in cerebral blood flow, another known effect of SNP, could also facilitate antipsychotic delivery to hypoperfused areas.^51^

For diuretics, the results were more nuanced. While a modest improvement in total symptom severity was observed, pooled effects for specific subscales (positive, negative, and general symptoms) did not reach statistical significance. Notably, this drug class included three mechanistically distinct agents: bumetanide,^45^ spironolactone,^44^, ^46^ and diazoxide.^43^ Bumetanide may exert neuropsychiatric effects by inhibiting the NKCC1 cotransporter, altering intracellular chloride gradients, and restoring GABAergic inhibition.^52^, ^53^ Spironolactone has been proposed to inhibit the NRG1–ERBB4 signaling pathway, which has been implicated in schizophrenia pathophysiology.^52^, ^54^, ^55^, ^56^, ^57^ Diazoxide, a potassium channel opener with diuretic properties (related to thiazide diuretics),^43^ may act *via* distinct mechanisms by regulating neuronal excitability and neurotransmitter release.^58^ Although the limited number of studies and small sample sizes preclude firm conclusions about individual agents, the observed trends suggest that certain diuretics—particularly those with non‐overlapping mechanisms—may merit further investigation as adjunctive treatments.

Only one study investigated the ARB telmisartan, an agent with both antihypertensive and PPAR‐γ agonist activity.^59^ While the effect did not reach statistical significance, negative SMDs were evidenced for PANSS subscales and total score scores, indicating a uniform, albeit modest, direction of effect across all symptom domains. These findings align with preclinical evidence suggesting neuroprotective effects of ARBs through renin–angiotensin system modulation,^60^ improved cerebral perfusion,^61^, ^62^ and anti‐inflammatory effects.^63^, ^64^, ^65^ Telmisartan has also been associated with reduced kynurenic acid levels and improved endothelial function,^66^ which might contribute to symptom improvement in schizophrenia. Though preliminary, these results warrant further studies, particularly in patients with comorbid hypertension or vascular risk factors.

Similarly, a single trial assessed the α_2_‐adrenergic agonist clonidine, and showed modest trends toward improvement in N‐PANSS, G‐PANSS, and T‐PANSS. The P‐PANSS, however, showed a near‐null effect with a wide 95% CI, suggesting substantial uncertainty. Clonidine's hypothesized benefit stems from noradrenergic modulation, which has been implicated in both positive and negative symptomatology.^67^, ^68^, ^69^ However, given the small sample and reliance on a single trial, definitive conclusions cannot be made.

Overall, our findings suggest that AHTs may exert beneficial effects on schizophrenia symptoms beyond their cardiovascular role. One plausible hypothesis is that improved cerebral perfusion (shared across several AHT classes) might enhance the delivery or efficacy of concurrently administered antipsychotic medications, particularly in brain regions characterized by hypoperfusion. Other potential mechanisms include the modulation of neuroinflammatory pathways, neurovascular function, and neurotransmitter systems. However, these remain speculative, and none of the included trials directly assessed such pathways. Furthermore, most studies did not report participants' baseline hypertension status, making it difficult to determine whether blood pressure control mediated any of the observed benefits. The convergence of effects across different AHT classes is intriguing but warrants cautious interpretation and underscores the need for further mechanistic research.

Safety outcomes were generally reassuring. Across nine studies, no significant increase in the incidence of AEs was detected for AHTs compared to placebo (*P =* 0.277). Interestingly, subgroup analysis suggested a lower incidence of AEs (*P =* 0.002) specifically among patients treated with SNP. SNP infusions also did not significantly affect systolic blood pressure, diastolic blood pressure, or heart rate, alleviating concerns about acute cardiovascular risks. However, considerable heterogeneity was observed in vital signs, likely reflecting variability in physiological responses among participants to dose titration protocols, baseline autonomic tone, and patient‐specific cardiovascular reactivity.

Similarly, diuretics alone demonstrated a numerically reduced AE incidence compared to placebo, though this association was not statistically significant (*P =* 0.120), likely due to the limited sample size and wide CIs. Collectively, these findings suggest a generally acceptable safety profile for the evaluated AHT classes in the context of adjunctive schizophrenia treatment. However, substantial methodological variability was noted in AE reporting across studies, ranging from standardized assessment tools to purely descriptive clinical monitoring. This variability could potentially limit the precision of safety conclusions, emphasizing the need for standardized AE reporting in future research and warranting further investigation in larger and longer trials with standardized safety assessments.

Several limitations should be acknowledged for this study. The total sample size of 436 participants remains modest, and most studies had short follow‐up periods. Heterogeneity in study design, drug class, and outcome reporting limits the generalizability of the results. The lack of patient‐level data precluded sex‐stratified analyses, preventing the examination of potential differences in treatment effects between male and female participants, as well as subgroup analyses based on other clinical features. Moreover, some data required estimation from medians and ranges using standard methods that assume symmetrical distributions and no correlation. Although these approaches are widely used, their accuracy may be reduced in small samples (*n* < 25), potentially introducing additional uncertainty.^33^ While publication bias was not detected, the small number of studies per subgroup limited statistical power to detect it reliably.

Additionally, IRR calculations assumed equal exposure times across treatment and placebo arms. This assumption may not hold for interventions with substantially different durations of administration, such as a single 4‐h SNP infusion compared with multi‐week oral regimens, which could affect the comparability of AE rates. Although we conducted drug‐class subgroup analyses to partially address this issue by grouping interventions with more similar exposure profiles, the absence of consistent person‐time or exposure‐adjusted AE data across trials prevented more precise analyses. Furthermore, the decision to restrict our search to studies published from 2000 onward may have introduced a potential time bias, where relevant studies conducted before this period, even if methodologically sound, would have been excluded. Similarly, limiting the search to English‐language publications may have introduced language bias, as potentially relevant trials published in other languages were not considered.

Notwithstanding the presence of studies with “some concerns” or “high” risk of bias, the consistency of results across different sensitivity analyses supports the robustness of the main findings. The leave‐one‐out analysis showed no single study unduly influenced the overall results across PANSS domains. Similarly, the exclusion of lower‐quality studies in the low‐risk‐only analysis did not materially alter the effect estimates or heterogeneity, suggesting minimal influence from potential bias. Together, these findings strengthen confidence in the treatment effects observed.

In conclusion, this meta‐analysis suggests that certain AHTs—particularly SNP—may offer adjunctive benefits for patients with schizophrenia, with the most consistent effects observed for negative symptoms. However, these preliminary results should be interpreted with caution given the modest effect sizes, small samples, and methodological heterogeneity. Larger, well‐designed trials with longer follow‐up are needed to validate these observations and clarify their clinical relevance, particularly in patients with treatment‐resistant symptoms.

## Funding

This research was supported by FEDER/Ministerio de Ciencia, Innovación y Universidades‐Agencia Estatal de Investigación, under grant PID2023‐147425OB‐I00.

## Disclosure statement

The authors have no competing interests to disclose.

## Author contributions

Conceptualization: TC, MB, JMM, FC, and SV. Data curation: TC, MB, and JV. Formal analysis: TC and JV. Investigation: TC, MB, JV, DR, JMM, FC, and SV. Methodology: TC and SV. Project administration: TC, SV, and FC. Software: TC and JV. Supervision: SV and FC. Validation: JV. Visualization: TC. Writing original draft preparation: TC, FC, and SV. All authors agree to be accountable for all aspects of the work. All authors have read and agreed to the submitted version of the manuscript.

## Supporting information


**Data S1:** Supporting Information.

## Data Availability

The study protocol (in Spanish), data used for all analyses, and analytic code are available from the corresponding author upon reasonable request.
