# Supplementary material for: Repurposed antihypertensive drugs for negative symptoms in schizophrenia: A systematic review and meta‐analysis
Source: Psychiatry Clin Neurosci. 2025 Sep 9;79(12):784–800. doi: 10.1111/pcn.13892 (PMC12683620; doi:10.1111/pcn.13892)
Supplement: Supplementary file 1 — Data S1: Supporting Information. [file PCN-79-784-s001.docx]

**Supplemental Material**

**Table of Contents**

[**Supplementary Tables** 2](#_Toc202211272)

[**Supplementary Table 1**. Keywords and Number of Results in the PubMed Search 2](#_Toc202211273)

[**Supplementary Table 2.** Keywords and Number of Results in the Web of Science Search 3](#_Toc202211274)

[**Supplementary Table 3.** Keywords and Number of Results in the Scopus Search 4](#_Toc202211275)

[**Supplementary Table 4.** Definitions of Adverse Events in the Studies Included in the Incidence Rate Ratio Meta-Analysis 5](#_Toc202211276)

[**Supplementary Table 5.** Main Causes for Excluding Screened Studies 6](#_Toc202211277)

[**Supplementary Table 6.** Eligibility Criteria Specific To Each Selected Study 8](#_Toc202211278)

[**Supplementary Table 7.** Summary of PANSS-Related Outcomes 12](#_Toc202211279)

[**Supplementary Table 8.** Egger’s Regression-Based Test 13](#_Toc202211280)

[**Supplementary Table 9.** P-value Corrections for Multiple Comparisons between PANSS Domains 14](#_Toc202211281)

[**Supplementary Table 10.** P-value Corrections for Multiple Comparisons between the IRR of Adverse Events and between Vital Signs during SNP Infusion 15](#_Toc202211282)

[**Supplementary Figures** 16](#_Toc202211283)

[**Supplementary Figure 1.** Forest Plots of the Effects of Antihypertensive Drugs on PANSS Domains on Low-Risk Studies 16](#_Toc202211284)

[**Supplementary Figure 2.** Funnel Plots 18](#_Toc202211285)

[**References** **(for the Supplemental Material)** 19](#_Toc202211286)

# **Supplementary Tables**

# **Supplementary Table 1**. Keywords and Number of Results in the PubMed Search

|  | **Keywords** |
| --- | --- |
| **Treatments** | (("double blind" OR "randomized controlled trial" OR RCT)  AND ("antihypertensive" OR "blood pressure medication" OR "ACE inhibitor" OR "angiotensin receptor antagonist" OR "beta blocker" OR "calcium channel blocker" OR "diuretic" OR "nitric oxide donor" OR "alpha 1 blocker" OR "alpha 2 agonist" OR benazepril OR captopril OR enalapril OR fosinopril OR imidapril OR lisinopril OR perindopril OR quinapril OR ramipril OR trandolapril OR candesartan OR eprosartan OR irbesartan OR losartan OR olmesartan OR telmisartan OR valsartan OR atenolol OR bisoprolol OR carvedilol OR nebivolol OR metoprolol OR labetalol OR propranolol OR esmolol OR nifedipine OR amlodipine OR manidipine OR barnidipine OR diltiazem OR felodipine OR lacidipine OR lercanidipine OR nitrendipine OR nicardipine OR verapamil OR spironolactone OR eplerenone OR furosemide OR indapamide OR amiloride OR torasemide OR xipamide OR bumetanide OR chlortalidone OR triamterene OR hydrochlorothiazide OR nitroglycerin OR isosorbide OR "glyceryl trinitrate" OR "sodium nitroprusside" OR prazosin OR doxazosin OR terazosin OR alfuzosin OR clonidine OR methyldopa OR guanfacine OR guanabenz) |
| **Disease** | AND (schizophrenia OR "schizoaffective disorder")) |
| **Timeframe** | AND ("2000/01/01"[Date - Publication] : "2025/03/31"[Date - Publication]) |
| **Results** | 39 |

# **Supplementary Table 2.** Keywords and Number of Results in the Web of Science Search

|  | **Keywords** |
| --- | --- |
| **Treatments** | TS=("double blind" OR "randomized controlled trial" OR RCT) AND TS=("antihypertensive" OR "blood pressure medication" OR "ACE inhibitor" OR "angiotensin receptor antagonist" OR "beta blocker" OR "calcium channel blocker" OR "diuretic" OR "nitric oxide donor" OR "alpha 1 blocker" OR "alpha 2 agonist" OR benazepril OR captopril OR enalapril OR fosinopril OR imidapril OR lisinopril OR perindopril OR quinapril OR ramipril OR trandolapril OR candesartan OR eprosartan OR irbesartan OR losartan OR olmesartan OR telmisartan OR valsartan OR atenolol OR bisoprolol OR carvedilol OR nebivolol OR metoprolol OR labetalol OR propranolol OR esmolol OR nifedipine OR amlodipine OR manidipine OR barnidipine OR diltiazem OR felodipine OR lacidipine OR lercanidipine OR nitrendipine OR nicardipine OR verapamil OR spironolactone OR eplerenone OR furosemide OR indapamide OR amiloride OR torasemide OR xipamide OR bumetanide OR chlortalidone OR triamterene OR hydrochlorothiazide OR nitroglycerin OR isosorbide OR "glyceryl trinitrate" OR "sodium nitroprusside" OR prazosin OR doxazosin OR terazosin OR alfuzosin OR clonidine OR methyldopa OR guanfacine OR guanabenz) |
| **Disease** | AND TS=(schizophrenia OR "schizoaffective disorder") |
| **Timeframe** | AND PY=(2000-2025) |
| **Results** | 157 |

# **Supplementary Table 3.** Keywords and Number of Results in the Scopus Search

|  | **Keywords** |
| --- | --- |
| **Treatments** | (TITLE-ABS-KEY("double blind" OR "randomized controlled trial" OR RCT) AND TITLE-ABS-KEY("antihypertensive" OR "blood pressure medication" OR "ACE inhibitor" OR "angiotensin receptor antagonist" OR "beta blocker" OR "calcium channel blocker" OR "diuretic" OR "nitric oxide donor" OR "alpha 1 blocker" OR "alpha 2 agonist" OR benazepril OR captopril OR enalapril OR fosinopril OR imidapril OR lisinopril OR perindopril OR quinapril OR ramipril OR trandolapril OR candesartan OR eprosartan OR irbesartan OR losartan OR olmesartan OR telmisartan OR valsartan OR atenolol OR bisoprolol OR carvedilol OR nebivolol OR metoprolol OR labetalol OR propranolol OR esmolol OR nifedipine OR amlodipine OR manidipine OR barnidipine OR diltiazem OR felodipine OR lacidipine OR lercanidipine OR nitrendipine OR nicardipine OR verapamil OR spironolactone OR eplerenone OR furosemide OR indapamide OR amiloride OR torasemide OR xipamide OR bumetanide OR chlortalidone OR triamterene OR hydrochlorothiazide OR nitroglycerin OR isosorbide OR "glyceryl trinitrate" OR "sodium nitroprusside" OR prazosin OR doxazosin OR terazosin OR alfuzosin OR clonidine OR methyldopa OR guanfacine OR guanabenz) |
| **Disease** | AND TITLE-ABS-KEY(schizophrenia OR "schizoaffective disorder")) |
| **Timeframe** | 2000 to Present |
| **Results** | 349 |

# **Supplementary Table 4.** Definitions of Adverse Events in the Studies Included in the Incidence Rate Ratio Meta-Analysis

| **Study** | **AE Definition/Assessment Tool** |
| --- | --- |
| Hallak et al. (2013) | UKU rating scale; AIMS; hourly monitoring of vitals. |
| Stone et al. (2016) | No formal definition; descriptive reporting. |
| Wang et al. (2018) | No formal definition; descriptive reporting. |
| Brown et al. (2019) | AIMS and SAFTEE. |
| Weiser et al. (2020) | No formal definition; descriptive reporting. |
| Nani et al. (2024) | UKU rating scale. |
| Zandifar et al. (2021) | AE checklist. |
| Hasan et al. (2023) | CTCAE v4.0 |
| Kruiper et al. (2023) | No formal definition; descriptive reporting. |
| Fan et al. (2017) | No formal definition; descriptive reporting. |

AE, adverse event; UKU, Udvalg for Kliniske Undersøgelser; AIMS, Abnormal Involuntary Movement Scale; SAFTEE, Systematic Assessment for Treatment Emergent Effects; CTCAE, Common Terminology Criteria for Adverse Events.

# **Supplementary Table 5.** Main Causes for Excluding Screened Studies

| **Excluded Study** | **Main cause for exclusion** |
| --- | --- |
| *Cheine et al. (2000) ^1^* | Systematic review. |
| *Keller et al. (2003) ^2^* | Review. |
| *Dolder et al. (2005) ^3^* | Observational study. |
| *Lemonnier et al. (2016) ^4^* | Case report. |
| *Kharod et al. (2019) ^5^* | Review. |
| *Hasan et al. (2020) ^6^* | Study protocol. |
| *Colbourne et al. (2021) ^7^* | Observational study. |
| *Lintunen et al. (2021) ^8^* | Observational study. |
| *van Dyke et al. (2018) ^9^* | Secondary analysis of an existing study (CATIE), not a stand-alone trial.. |
| *Carnovale et al. (2023) ^10^* | Review. |
| *Oh and Fan (2019) ^11^* | Review. |
| *Stone et al. (2017) ^12^* | Reply to a letter to the editor. |
| *Zoupa and Pitsikas (2021) ^13^* | Review. |
| *Maia de Oliveira et al. (2016) ^14^* | Review. |
| *Fei et al. (2023) ^15^* | Protocol for a systematic review. |
| *Winder-Rhodes et al. (2010) ^16^* | No diagnosis of schizophrenia: study conducted with healthy volunteers |
| *Hajjar et al. (2013) ^17^* | No confirmed schizophrenia diagnosis: participants had hypertension and executive dysfunction. |
| *Rezende et al (2017) ^18^* | No diagnosis of schizophrenia: conducted in healthy participants using a ketamine challenge model, not in patients with schizophrenia. |
| *Merrit et al. (2020) ^19^* | No confirmed schizophrenia diagnosis: participants had first-episode psychosis not yet treated with antipsychotics. Additionally, glyceryl trinitrate is primarily used as a vasodilator in cardiovascular conditions, rather than as an antihypertensive. |
| *Zandifar et al. (2023) ^20^* | No diagnosis of schizophrenia: participants had bipolar disorder. |
| *Adelino et al. (2024) ^21^* | No confirmed schizophrenia diagnosis: drug-naïve first-episode psychosis patients. |
| *Oranje and Glenthøj (2013) ^22^* | No actual PANSS values: no means or mean changes in PANSS scores were provided. |
| *Maia de Oliveira et al. (2015) ^23^* | Did not report any PANSS outcomes. |
| *Rahmanzadeh et al. (2016) ^24^* | Did not report PANSS outcomes: focused exclusively on hallucinations (PANSS-P3) in treatment-resistant patients; no cognitive or general symptom outcomes assessed. |
| *Fan et al. (2019) ^25^* | Did not report any PANSS outcomes: primary and secondary endpoints focused exclusively on metabolic parameters |
| *Burdick et al. (2020) ^26^* | PANSS outcomes not reported. |
| *Vahdani et al. (2020) ^27^* | PANSS outcomes only reported at baseline. |
| *Adelino et al. (2021) ^28^* | Unclear outcome reporting: overlapping group plots and no indication of whether values represent means or medians; error bars not labeled (CI, SD, or SE) |
| *Guimaraes et al. (2021) ^29^* | Unclear outcome reporting: inconsistencies between PANSS values reported in tables and figures. Additionally, mononitrate isosorbide is primarily used as a vasodilator in cardiovascular conditions, rather than as an antihypertensive |
| *Egan et al. (2013) ^30^* | No AHT: MK-8998 is a T-type calcium channel antagonist that has been specifically developed as a central nervous system agent, which is not classified as an antihypertensive drug. |

# **Supplementary Table 6.** Eligibility Criteria Specific To Each Selected Study

| **Study (year)** | **Inclusion Criteria** | **Exclusion Criteria** |
| --- | --- | --- |
| Hallak et al. (2013) | - 18 to 65 years of age - Diagnosis of schizophrenia using DSM-IV - Within first 5 years of diagnosis - Competent and willing to give informed consent - Able to complete the required evaluations - Female participants willing to have a pregnancy test before treatment - Patients in an acute psychotic episode requiring full-time hospitalization according to clinical referral by the relevant mental health service | - Relevant medical illness (renal, hepatic, or cardiac) in the opinion of the investigators - Prior history of intolerance to sodium nitroprusside - Presence of a seizure disorder, not including clozapine-induced seizures - Currently taking clozapine - Any change of psychotropic medications within the previous 6 weeks - Diagnosis of substance abuse (except nicotine or caffeine) or dependence within the last 3 months according to DSM-IV criteria - Pregnant or breastfeeding - Used illicit substances or alcohol in the past 3 months - History of any major medical illness |
| Stone et al. (2016) | - Capacity to give informed consent - 18–60 years of age - Patients with a diagnosis of schizophrenia or schizoaffective disorder according to DSM-IV - Currently experiencing an exacerbation of symptoms (score > 20 for PANSS Positive subscale) - Currently taking antipsychotics | - Prior history of intolerance to sodium nitroprusside - Presence of a seizure disorder - Any change in psychotropic medication in previous 6 weeks - Diagnosis of substance abuse - Pregnancy (as determined by urine test) or breastfeeding - Relevant medical history including untreated hypothyroidism, hyponatremia, ischemic heart disease, impaired cerebral circulation, renal impairment, vitamin B12 deﬁciency or Leber’s optic atrophy |
| Wang et al. (2018) | - Diagnosis of schizophrenia according to DSM-IV - Age between 18 and 45 years - Han ethnicity - Competent and willing to provide informed consent - Able to complete the required evaluations - Females willing to undergo a pregnancy test before treatment - Experiencing an acute psychotic episode with a PANSS score ≥ 70 - Currently taking antipsychotic medication | - DSM-IV diagnosis of:   - Alcohol or substance abuse within the past month   - Alcohol or substance dependence within the past 6 months - Recent use of street drugs (confirmed by urine toxicology test) - History of seizures or head trauma with loss of consciousness, resulting cognitive sequelae or need for rehabilitation - Pregnancy or breastfeeding - Presence of any relevant medical illness, including:   - Untreated hypothyroidism   - Hyponatremia   - Ischemic heart disease   - Hypotension   - Impaired cerebral circulation   - Renal impairment   - Vitamin B12 deficiency   - Leber's optic atrophy - Prior history of intolerance to sodium nitroprusside - Any change in psychotropic medication within the past 6 weeks - History of any major medical illness |
| Brown et al. (2019) | - Total PANSS score ≥ 70 - Score of ≥ 4 on two or more of the following PANSS items: Delusions; conceptual disorganization; hallucinatory behavior; suspiciousness; unusual thought content - CGI-S score ≥ 4 - Diagnosis and symptom severity confirmed by an independent expert remote rater - Ongoing antipsychotic medication treatment for at least 8 weeks - Stable antipsychotic dose for at least 4 weeks - In the past year, failure to achieve clinically significant symptom reduction after at least 8 weeks of treatment with at least one antipsychotic at a therapeutic dose - Treatment history confirmed using the *Massachusetts General Hospital Fast Additive Summary of Treatment*. | - Any major medical illness - Symptomatic orthostatic hypotension - Treatment with medications that either interfere with the metabolism or excretion of SNP, have known drug interactions with SNP, or could pose a significant health risk to participants - Current alcohol or substance use disorders (excluding nicotine) - Pregnancy or breastfeeding - Imminent risk of suicide or harm to self or others |
| Weiser et al. (2020) | - Identical to Hallak et al. (2013) | - Identical to Hallak et al. (2013) |
| Nani et al. (2024) | - Diagnosis of SCZ according to DSM-IV-TR criteria - Antipsychotic treatment without changes for at least 6 months - Age between 18-45 years for both sexes - Being refractory or super-refractory to classical antipsychotic treatment - Being able to provide written informed consent | - Presence of relevant medical condition (such as cardiovascular diseases, primary neurological diseases, renal failure, liver disease, thyroid diseases, hyponatremia, hypovitaminosis, pregnancy or breastfeeding) - Diagnosis of abuse or dependence of illicit psychoactive substances according to DSM-IV-TR criteria - History of hypersensitivity to SNP - Inability to provide written informed consent |
| Akhondzadeh et al. (2002) | - Inpatients diagnosed with chronic schizophrenia by DSM-IV criteria - Age ≥ 18 years old - Minimum PANSS total score ≥ 60 - No neuroleptics for 1 week prior to entry - No depot neuroleptics for at least 2 months before the study | - Clinically significant organic or neurological disorders - Other serious psychotic disorders aside from schizophrenia - Serious cardiovascular problems, including recent myocardial infarction, hypotension, diabetes mellitus - Pregnancy or lactation - Women of reproductive age without adequate contraception |
| Rahmanzadeh et al. (2016) | - Diagnosis of schizophrenia (any subtype) according to DSM-IV | - PANSS total score < 65 - Documented fluctuation in symptoms - Concurrent significant psychiatric disorder (other than schizophrenia) - Significant organ disorders (any type) |
| Zandifar et al. (2021) | - Adults aged 18–60 years - Diagnosis of schizophrenia per DSM-5 criteria - Minimum 2 years disease duration (i.e., chronic schizophrenia) - Diagnosis confirmed via SCID-5-CV by an experienced psychiatrist - PANSS total score ≥ 60 - PANSS negative subscale score ≥ 19 | - HDRS ≥ 14 (17-item version) and/or PANSS depression item ≥ 4 - Current or past 6-month history of alcohol/substance dependence - IQ < 70 - Presence of other mental disorders - Severe medical condition - Pregnancy or lactation - Women of childbearing age without adequate contraception - Electroconvulsive therapy in the last 6 months - Drug- or insulin-dependent diabetes - Hepatitis - Congestive heart failure - Use of neuroleptics within the last 7 days - Use of long-acting neuroleptics within the last 30 days |
| Hasan et al. (2023) | - Inpatients and outpatients (men and women) aged 18–65 years - Primary diagnosis of schizophrenia according to ICD-10, confirmed by the MINI - Able to provide informed consent - Receiving stable antipsychotic treatment for at least 1 week - Treated with no more than two antipsychotics - PANSS total score ≥ 75 - Illness duration of ≥ 6 months - Female participants with a negative serum pregnancy test at baseline and using a medically approved method of contraception | - Incapacity to give informed consent - Suicidality or danger to others - Severe somatic or neurological comorbidities - Non-compliance history interfering with study participation - Current treatment with clozapine or an antipsychotic with exclusive renal elimination (e.g., amisulpride) - Planned initiation of antidepressants or mood stabilizers during the intervention - Prior treatment allowed except with lithium or renal-eliminated antidepressants - Substance dependency (excluding tobacco or caffeine) in the past 6 months - History of seizures (only relevant for the TMS physiological substudy) - Documented intolerance to spironolactone or placebo capsules - Acute kidney failure, anuria, or severe kidney insufficiency: - Creatinine clearance < 30 ml/min/1.73 m² - Serum creatinine > 1.8 mg/dL - Clinically relevant hyperkalemia or hyponatremia - Clinically relevant hypotension (Syst./Diast. < 100/80 mmHg) - Simultaneous use of potassium-sparing diuretics, ACE inhibitors, ARBs, NSAIDs, thiazide diuretics, carbenoxolone, digoxin, or neomycin - Coercive treatment - Treatment-resistant or treatment-naïve schizophrenia - Insufficient understanding of German - Pregnancy - Absence of a safe and approved method of contraception |
| Fan et al. (2017) | - Adults (18–65 years) diagnosed with schizophrenia or schizoaffective disorder - Recruited from an urban community-based general mental health clinic (Boston, MA) - Ongoing treatment with clozapine or olanzapine for at least 6 months - Stable dose of current antipsychotic for at least 1 month - English-speaking and able to complete cognitive assessments | - Inability to provide informed consent - Current substance use - Unstable medical conditions - Current insulin treatment for diabetes - History of immunosuppression - Current or recent radiation or chemotherapy for cancer - Chronic steroid use - Pregnancy or breastfeeding - Use of medications that may interact with telmisartan, including diuretics, ACE inhibitors, spironolactone, potassium supplements, digoxin, and warfarin. |
| Kruiper et al. (2023) | - Age between 18 and 55 years - Ongoing treatment with antipsychotic medication - Diagnosis of schizophrenia or schizoaffective disorder confirmed using the MINI - Assessment of current symptomatology using the PANSS - Physical examination confirming good physical health | - Total PANSS score < 55 - Presence of any medical condition contra-indicated for clonidine treatment - Pre-existing orthostatic hypotension - Supine blood pressure <85 mmHg - Supine heart rate <50 bpm - Severe brady-arrhythmias - Use of beta-blockers or mirtazapine - Pregnancy or breastfeeding |

ACE, angiotensin-converting enzyme; ARB, angiotensin II receptor blocker; CGI-S, Clinical Global Impression–Severity scale; DSM-IV / DSM-IV-TR / DSM-5, Diagnostic and Statistical Manual of Mental Disorders, Fourth Edition / Text Revision / Fifth Edition; HDRS, Hamilton Depression Rating Scale; ICD-10, International Classification of Diseases, 10th Revision; IQ, intelligence quotient; MINI, Mini-International Neuropsychiatric Interview; NSAID, nonsteroidal anti-inflammatory drug; PANSS, Positive and Negative Syndrome Scale; SCID, Structured Clinical Interview for DSM Disorders; SCID-5-CV, Structured Clinical Interview for DSM-5 – Clinical Version; SNP, sodium nitroprusside; TMS, transcranial magnetic stimulation.

# **Supplementary Table 7.** Summary of PANSS-Related Outcomes

|  |  | **Mean (SD) N-PANSS** | | **Mean (SD) P-PANSS** | | **Mean (SD) G-PANSS** | | **Mean (SD) T-PANSS** | |
| --- | --- | --- | --- | --- | --- | --- | --- | --- | --- |
| **Study (Year)** | **Groups** | **Baseline** | **EoT** | **Baseline** | **EoT** | **Baseline** | **EoT** | **Baseline** | **EoT** |
| Akhondzadeh et al. (2002) | Diazoxide | 24.1 (5.8) | 15.0 (4.2) | 25.3 (7.6) | 10.9 (2.5) | 44.3 (6.2) | 23.6 (3.8) | 94.1 (11.5) | 49.1 (5.9) |
|  | Pb | 24.0 (3.6) | 15.6 (4.1) | 26.0 (4.1) | 13.1 (3.6) | 44.8 (4.3) | 26.8 (4.5) | 94.8 (7.3) | 58.0 (8.9) |
| Hallak et al. (2013) | SNP | 29.1 (2.3) | 21.9 (1.9) | NR | NR | NR | NR | NR | NR |
|  | Pb | 29.4 (2.1) | 28.2 (2.1) | NR | NR | NR | NR | NR | NR |
| Stone et al. (2016) | SNP | 21.4 (6.3) | 18.8 (6.6) | 22.3 (1.8) | 20.0 (2.5) | 37.0 (9.7) | 34.5 (10.2) | NR | NR |
|  | Pb | 20.1 (6.6) | 18.2 (6.1) | 23.6 (2.5) | 21.7 (4.0) | 34.5 (8.4) | 32.0 (8.0) | NR | NR |
| Rahmanzadeh et al. (2016) | Bumetanide | 17.4 (6.9) | 17.2 (6.6) | 18.2 (4.2) | 18.0 (6.3) | 14.0 (4.0) | 13.1 (3.6) | 68.3 (15.7) | 67.8 (15.8) |
|  | Pb | 20.3 (9.0) | 20.6 (8.7) | 17.5 (7.6) | 18.1 (7.2) | 15.8 (5.9) | 14.6 (3.8) | 75.4 (14.8) | 75.4 (14.3) |
| Fan et al. (2017) | Telmisartan | 19.9 (6.5) | NR | 16.7 (5.5) | NR | 34.6 (6.8) | NR | 71.2 (12.1) | NR |
|  | Pb | 20.0 (5.5) | NR | 16.4 (7.6) | NR | 34.1 (10.0) | NR | 70.5 (20.4) | NR |
| Wang et al. (2018) | SNP | 23.0 (2.6) | 20.3 (4.5) | 20.5 (2.7) | 17.7 (3.2) | 34.5 (7.2) | 32.7 (6.5) | NR | NR |
|  | Pb | 24.4 (2.5) | 23.2 (3.3) | 21.3 (2.9) | 19.0 (2.6) | 32.4 (2.7) | 32.0 (3.0) | NR | NR |
| Brown et al. Phase I (2019) | SNP | 20.9 (5.5) | 20.9 (4.8) | 24.9 (3.5) | 23.1 (4.6) | NR | NR | 83.6 (10.0) | 77.6 (12.2) |
|  | Pb | 19.1 (4.7) | 19.8 (5.1) | 22.5 (3.2) | 21.3 (3.6) | NR | NR | 79.8 (8.9) | 76.7 (11.0) |
| Brown et al. Phase II (2019) | SNP | 18.5 (5.8) | 20.8 (4.4) | 20.7 (3.6) | 21.3 (4.6) | NR | NR | 74.0 (12.0) | 71.8 (10.6) |
|  | Pb | 18.1 (6.2) | 19.8 (4.7) | 21.8 (3.6) | 22.4 (3.5) | NR | NR | 78.8 (10.1) | 74.7 (9.0) |
| Weiser et al. (2020) | SNP | 21.2 (2.7) | 16.5 (3.9) | 18.6 (4.0) | NR | 41.5 (3.6) | NR | 81.3 (6.8) | 65.1 (10.9) |
|  | Pb | 19.8 (3.1) | 18.0 (3.3) | 18.0 (3.6) | NR | 41.1 (3.2) | NR | 78.9 (7.3) | 71.1 (9.1) |
| Zandifar et al. (2021) | Spironolactone | 25.0 (5.4) | 11.5 (2.9) | 34.0 (4.9) | 20.2 (3.0) | 43.0 (6.7) | 21.0 (8.6) | 101.9 (8.2) | 54.4 (9.2) |
|  | Pb | 25.5 (5.6) | 16.9 (3.6) | 35.6 (2.7) | 25.5 (4.2) | 40.9 (9.7) | 21.3 (11.7) | 102.0 (10.9) | 63.6 (11.1) |
| Hasan et al. (2023) | Spironolactone 100 mg | 14.2 (2.3) | 13.2 (3.3) | 11.9 (1.9) | 10.9 (3.2) | 27.0 (3.3) | 23.3 (4.1) | 88.9 (9.8) | 78.7 (15.0) |
|  | Spironolactone 200 mg | 14.2 (2.2) | 11.9 (2.3) | 12.0 (1.6) | 8.6 (1.2) | 25.0 (2.7) | 22.2 (3.3) | 85.5 (9.4) | 70.9 (10.5) |
|  | Pb | 14.9 (1.8) | 13.5 (2.2) | 12.4 (1.8) | 10.2 (2.3) | 27.0 (3.1) | 24.1 (4.2) | 90.5 (9.6) | 80.0 (11.6) |
| Kruiper et al. (2023) | Clonidine | 17.8 (6.2) | 15.4 (4.6) | 15.4 (4.6) | 13.8 (4.4) | 32.1 (6.5) | 28.5 (4.6) | 66.3 (10.9) | 57.7 (9.9) |
|  | Pb | 19.6 (5.9) | 18.7 (5.8) | 21.4 (7.5) | 19.8 (6.6) | 34.6 (6.3) | 32.1 (7.2) | 75.6 (16.6) | 70.6 (16.2) |
| Nani et al. (2024) | SNP | 19.9 (7.1) | 15.9 (6.0) | 16.3 (4.0) | 10.1 (2.3) | NR | NR | NR | NR |
|  | Pb | 22.7 (4.3) | 20.7 (4.2) | 9.9 (3.3) | 7.7 (0.9) | NR | NR | NR | NR |

N-PANSS, positive and negative syndrome scale negative symptoms subscale score; P-PANSS, positive and negative syndrome scale positive symptoms subscale Score; G-PANSS, positive and negative syndrome scale general psychopathology subscale score; T-PANSS, positive and negative syndrome scale total score; SD, standard deviation; EoT, end of trial; NR, not reported; Pb, placebo; SNP, sodium nitroprusside.

# **Supplementary Table 8.** Egger’s Regression-Based Test

| **Outcome** | **Bias estimate (SE)** | **t-value** | ***df*** | **p-value** |
| --- | --- | --- | --- | --- |
| N-PANSS | -2.66 (1.53) | -1.74 | 11 | 0.110 |
| P-PANSS | -1.22 (1.74) | -0.70 | 10 | 0.499 |
| G-PANSS | 1.63 (1.09) | 1.48 | 9 | 0.173 |
| T-PANSS * | - | - | - | - |
| Adverse Events | 1.19 | 1.61 | 9 | 0.142 |

SE, standard error; df, degrees of freedom; N-PANSS, Positive and Negative Symptom Scale Negative Subscale Score; P-PANSS, Positive and Negative Symptom Scale Positive Subscale Score; G-PANSS, Positive and Negative Symptom General Psychopathology Subscale Score; T-PANSS, Positive and Negative Symptom Scale Total Score

Model: weighted regression with multiplicative dispersion.

Predictor: standard error.

* Not enough studies to perform the test.

# **Supplementary Table 9.** P-value Corrections for Multiple Comparisons between PANSS Domains

| **Outcome** | **Raw p-value** | **Holm’s Correction** |
| --- | --- | --- |
| **All AHTs** |  |  |
| N-PANSS | 0.001 | 0.003 |
| P-PANSS | 0.014 | 0.014 |
| G-PANSS | 0.007 | 0.013 |
| T-PANSS | <0.001 | 0.001 |
| **SNP Only** |  |  |
| N-PANSS | 0.028 | 0.085 |
| P-PANSS | 0.020 | 0.080 |
| G-PANSS | 0.039 | 0.085 |
| T-PANSS | 0.084 | 0.085 |
| **Diuretics Only** |  |  |
| N-PANSS | 0.167 | 0.500 |
| P-PANSS | 0.356 | 0.500 |
| G-PANSS | 0.207 | 0.500 |
| T-PANSS | 0.027 | 0.107 |

AHT, antihypertensive drugs; SNP, sodium nitroprusside; N-PANSS, Positive and Negative Symptom Scale Negative Subscale Score; P-PANSS, Positive and Negative Symptom Scale Positive Subscale Score; G-PANSS, Positive and Negative Symptom General Psychopathology Subscale Score; T-PANSS, Positive and Negative Symptom Scale Total Score

# **Supplementary Table 10.** P-value Corrections for Multiple Comparisons between the IRR of Adverse Events and between Vital Signs during SNP Infusion

| **Outcome** | **Raw p-value** | **Holm’s Correction** |
| --- | --- | --- |
| **All AHTs** |  |  |
| IRR of AEs | 0.277 | 0.284 |
| **SNP Only** |  |  |
| IRR of AEs | 0.002 | 0.006 |
| Vital Signs |  |  |
| Systolic BP | 0.632 | 1.000 |
| Diastolic BP | 0.754 | 1.000 |
| Heart Rate | 0.633 | 1.000 |
| **Diuretics Only** |  |  |
| IRR of AEs | 0.120 | 0.240 |

AHT, antihypertensive drugs; SNP, sodium nitroprusside; IRR, incidence rate ratio; AE, adverse event; BP, blood pressure.

# **Supplementary Figures**

# **Supplementary Figure 1.** Forest Plots of the Effects of Antihypertensive Drugs on PANSS Domains on Low-Risk Studies

|  |
| --- |
|  |
|  |
|  |

(A) Negative symptoms subscale (N-PANSS); (B) Positive symptoms subscale (P-PANSS); (C) General psychopathology subscale (G-PANSS); (D) Total PANSS score (T-PANSS). Negative values favor the experimental (Exp) group.

# **Supplementary Figure 2.** Funnel Plots


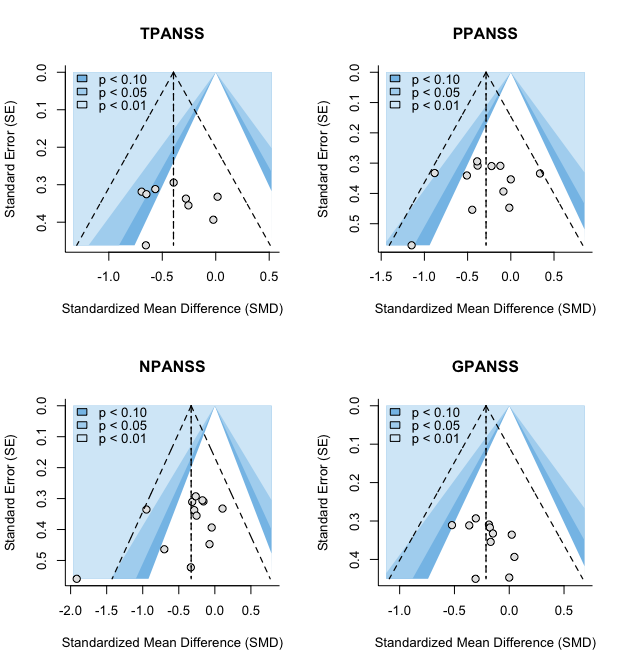


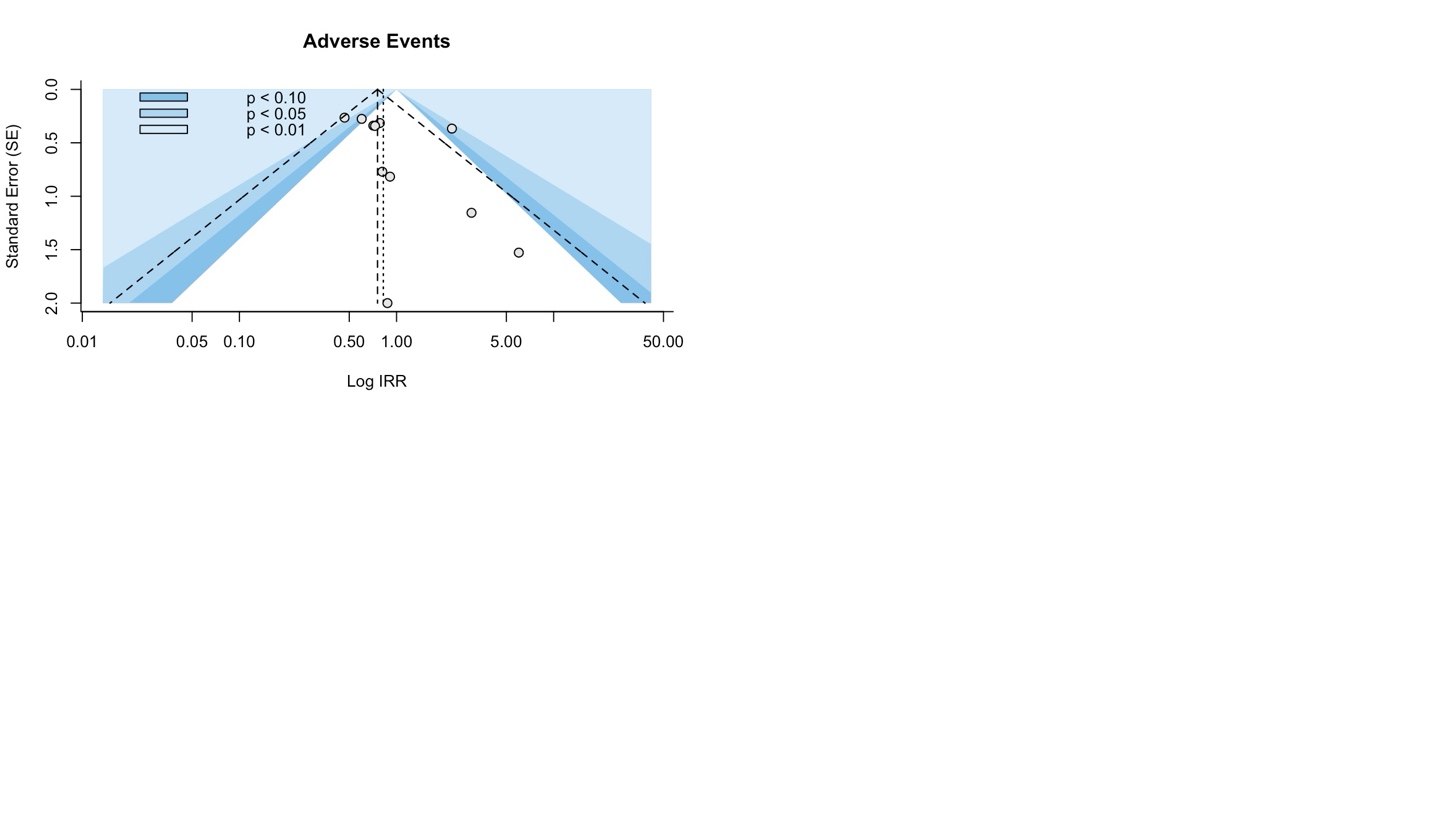


PANSS, Positive and Negative Syndrome Scale; TPANSS, Total PANSS Score; PPANSS, PANSS Positive Subscale Score; NPANSS, PANSS Negative Subscale Score; GPANSS, PANSS General Psychopathology Subscale Score; SMD, standardized mean difference.

# **References** **(for the Supplemental Material)**

1. Cheine M, Ahonen J, Wahlbeck K. Beta-blocker supplementation of standard drug treatment for schizophrenia. *Cochrane Database Syst Rev* 2000: Cd000234.

2. Keller S, Frishman WH. Neuropsychiatric effects of cardiovascular drug therapy. *Cardiol. Rev.* 2003; **11**: 73-93.

3. Dolder CR, Furtek K, Lacro JP, Jeste DV. Antihypertensive medication adherence and blood pressure control in patients with psychotic disorders compared to persons without psychiatric illness. *Psychosomatics* 2005; **46**: 135-141.

4. Lemonnier E, Lazartigues A, Ben-Ari Y. Treating Schizophrenia With the Diuretic Bumetanide: A Case Report. *Clin Neuropharmacol* 2016; **39**: 115-7.

5. Kharod SC, Kang SK, Kadam SD. Off-Label Use of Bumetanide for Brain Disorders: An Overview. *Front Neurosci* 2019; **13**: 310.

6. Hasan A, Roeh A, Leucht S et al. Add-on spironolactone as antagonist of the NRG1-ERBB4 signaling pathway for the treatment of schizophrenia: Study design and methodology of a multicenter randomized, placebo-controlled trial. *Contemp Clin Trials Commun* 2020; **17**: 100537.

7. Colbourne L, Luciano S, Harrison PJ. Onset and recurrence of psychiatric disorders associated with anti-hypertensive drug classes. *Transl Psychiatry* 2021; **11**: 319.

8. Lintunen J, Lahteenvuo M, Tiihonen J, Tanskanen A, Taipale H. Adenosine modulators and calcium channel blockers as add-on treatment for schizophrenia. *NPJ Schizophr* 2021; **7**: 1.

9. Van Dyke P, Thomas KL. Concomitant calcium channel blocker and antipsychotic therapy in patients with schizophrenia: Efficacy analysis of the CATIE-Sz phase 1 data. *Annals of Clinical Psychiatry* 2018; **30**: 6-16.

10. Carnovale C, Perrotta C, Baldelli S et al. Antihypertensive drugs and brain function: mechanisms underlying therapeutically beneficial and harmful neuropsychiatric effects. *Cardiovasc Res* 2023; **119**: 647-667.

11. Oh SJ, Fan X. Current understanding on the role of nitric oxide and therapeutic potential of NO supplementation in schizophrenia. *Schizophrenia Research* 2020; **222**: 23-30.

12. Stone J, Koychev I, Reilly T, McGuire P. Reply to: Letter to the Editor: Sodium nitroprusside for schizophrenia: could methodological variables account for the different results obtained? *Psychological Medicine* 2017; **47**: 983-983.

13. Zoupa E, Pitsikas N. The Nitric Oxide (NO) Donor Sodium Nitroprusside (SNP) and Its Potential for the Schizophrenia Therapy: Lights and Shadows. *Molecules* 2021; **26**.

14. Maia-de-Oliveira JP, Kandratavicius L, Nunes EA, Machado-de-Sousa JP, Hallak JE, Dursun SM. Nitric Oxide's Involvement in the Spectrum of Psychotic Disorders. *Current Medicinal Chemistry* 2016; **23**: 2680-2691.

15. Fei X, Wang S, Li J, Wang J, Gao Y, Hu Y. The efficacy and safety of sodium nitroprusside in the treatment of schizophrenia: Protocol for an updated systematic review and meta-analysis. *PLoS ONE* 2023; **18**.

16. Winder-Rhodes SE, Chamberlain SR, Idris MI, Robbins TW, Sahakian BJ, Mueller U. Effects of modafinil and prazosin on cognitive and physiological functions in healthy volunteers. *Journal of Psychopharmacology* 2010; **24**: 1649-1657.

17. Hajjar I, Hart M, Chen YL et al. Antihypertensive therapy and cerebral hemodynamics in executive mild cognitive impairment: results of a pilot randomized clinical trial. *J Am Geriatr Soc* 2013; **61**: 194-201.

18. Rezende TMN, Maia-de-Oliveira JP, Kandratavicius L et al. Effects of sodium nitroprusside in the prevention of schizophrenia-like symptoms induced by ketamine – A translational double-blind study. *Archives of Clinical Psychiatry (São Paulo)* 2017; **44**.

19. Merritt K, Catalan A, Cowley S et al. Glyceryl trinitrate in first-episode psychosis unmedicated with antipsychotics: A randomised controlled pilot study. *J Psychopharmacol* 2020; **34**: 839-847.

20. Zandifar A, Badrfam R, Gholamian F, Shafiee A. Efficacy of spironolactone as adjunctive therapy to sodium valproate in bipolar-I disorder: A double-blind, randomized, placebo-controlled clinical trial. *Brain and Behavior* 2023; **13**.

21. Adelino MPM, Nunes MV, Nunes MFQ, Quarantini LC, Hallak JEC, Lacerda ALT. Efficacy of sodium nitroprusside in the treatment of drug-naive subjects in first episode psychosis - An open label study. *Schizophrenia Research* 2024; **269**: 114-115.

22. Oranje B, Glenthøj BY. Clonidine normalizes sensorimotor gating deficits in patients with schizophrenia on stable medication. *Schizophr Bull* 2013; **39**: 684-91.

23. Maia-de-Oliveira JP, Abrao J, Evora PR et al. The effects of sodium nitroprusside treatment on cognitive deficits in schizophrenia: a pilot study. *J Clin Psychopharmacol* 2015; **35**: 83-5.

24. Rahmanzadeh R, Eftekhari S, Shahbazi A et al. Effect of bumetanide, a selective NKCC1 inhibitor, on hallucinations of schizophrenic patients; a double-blind randomized clinical trial. *Schizophr Res* 2017; **184**: 145-146.

25. Fan X, Copeland P, Nawras S et al. Adjunctive telmisartan treatment on body metabolism in clozapine or olanzapine treated patients with schizophrenia: a randomized, double blind, placebo controlled trial. *Psychopharmacology (Berl)* 2019; **236**: 1949-1957.

26. Burdick KE, Perez-Rodriguez M, Birnbaum R et al. A molecular approach to treating cognition in schizophrenia by calcium channel blockade: An open-label pilot study of the calcium-channel antagonist isradipine. *Schizophr Res Cogn* 2020; **21**: 100180.

27. Vahdani B, Armani Kian A, Esmaeilzadeh A, Zenoozian S, Yousefi V, Mazloomzadeh S. Adjunctive Raloxifene and Isradipine Improve Cognitive Functioning in Patients With Schizophrenia: A Pilot Study. *J Clin Psychopharmacol* 2020; **40**: 457-463.

28. Adelino MPM, Nunes MV, Nunes MFQ et al. Treatment-resistant schizophrenia - A RCT on the effectiveness of repeated-dose sodium nitroprusside. *Schizophr Res* 2021; **231**: 70-72.

29. Guimaraes TM, Guimaraes MRC, Oliveira IAF et al. Mononitrate Isosorbide as an Adjunctive Therapy in Schizophrenia: A Randomized Controlled Crossover Trial. *J. Clin. Psychopharmacol.* 2021; **41**: 260-266.

30. Egan MF, Zhao X, Smith A et al. Randomized controlled study of the T-type calcium channel antagonist MK-8998 for the treatment of acute psychosis in patients with schizophrenia. *Human Psychopharmacology-Clinical and Experimental* 2013; **28**: 124-133.
